# Age‐Dependent Bi‐Phasic Dynamics of Ly49^+^ CD8 ^+^ Regulatory T Cell Population

**DOI:** 10.1111/acel.14461

**Published:** 2024-12-18

**Authors:** Saranya Srinivasan, Shruti Mishra, Kenneth Ka‐Ho Fan, Liwen Wang, John Im, Courtney Segura, Neelam Mukherjee, Gang Huang, Manjeet Rao, Chaoyu Ma, Nu Zhang

**Affiliations:** ^1^ Department of Microbiology, Immunology and Molecular Genetics, Long School of Medicine University of Texas Health Science Center at San Antonio San Antonio Texas USA; ^2^ Gilead Sciences Inc California USA; ^3^ Department of Hematology, Third Xiangya Hospital Central South University Changsha Hunan Province China; ^4^ Department of Urology University of Texas Health Science Center at San Antonio San Antonio Texas USA; ^5^ Department of Cell Systems and Anatomy Greehey Children's Cancer Research Institute San Antonio Texas USA; ^6^ South Texas Veterans Health Care System San Antonio Texas USA

**Keywords:** aging, CD8 Treg, Helios, NKG2D

## Abstract

Aging is tightly associated with reduced immune protection but increased risk of autoimmunity and inflammatory conditions. Regulatory T cells are one of the key cells to maintaining immune homeostasis. The age‐dependent changes in CD4^+^Foxp3^+^ regulatory T cells (Tregs) have been well documented. However, the nonredundant Foxp3^−^CD8^+^ Tregs were never examined in the context of aging. This study first established clear distinctions between phenotypically overlapping CD8^+^ Tregs and virtual memory T cells. Then, we elucidated the dynamics of CD8^+^ Tregs across the lifespan in mice and further extended our investigation to human peripheral blood mononuclear cells (PBMCs). In mice, we discovered a bi‐phasic dynamic shift in the frequency of CD8^+^CD44^hi^CD122^hi^Ly49^+^ Tregs, with a steady increase in young adults and a notable peak in middle age followed by a decline in older mice. Transcriptomic analysis revealed that mouse CD8^+^ Tregs upregulated a selected set of natural killer (NK) cell‐associated genes, including NKG2D, with age. Importantly, NKG2D might negatively regulate CD8^+^ Tregs. Additionally, by analyzing a scRNA‐seq dataset of human PBMC, we found a distinct CD8^+^ Treg‐like subset (Cluster 10) with comparable age‐dependent frequency changes and gene expression, suggesting a conserved aging pattern in CD8^+^ Treg across mice and humans. In summary, our findings highlight the importance of CD8^+^ Tregs in immune regulation and aging.

## Graphical abstract

The biology of Ly49^+^CD8^+^ regulatory T cells (Tregs) during aging is unknown. Here, we demonstrate that CD8^+^ Tregs exhibit unique bi‐phasic population dynamics during aging, with a gradual increase until middle age followed by a progressive loss. This age‐dependent pattern is highly conserved between mice and humans.

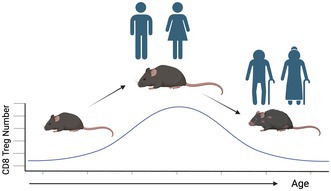

## 1 Introduction

Aging is a complex biological process that significantly affects the immune system. It is well established that aging is associated with a progressive decline in immune response, and increased risk of infection and cancer. In addition, aging is also characterized by chronic low‐grade inflammation called “inflammaging” (Borgoni et al. 2021; Franceschi et al. 2000) and surged frequency of autoimmune disorders. Immune aging exhibits distinct manifestations in different immune cell populations, for example, reduced naive/ memory T‐cell ratio and the accumulation of age‐associated B and T cells that may collectively act as critical contributors to autoimmune pathogenesis (Cancro 2020; Liu et al. 2023; Miller 2020; Nikolich‐Zugich 2018; Zhao et al. 2022). Thus, to fully understand immune aging, it is pivotal to elucidate the kinetics and molecular signatures of each immune cell subset during aging.

Accumulating evidence links many autoimmune disorders and cancer to age‐related accumulation and defects in CD4^+^ Tregs (Garg et al. 2014; Guo et al. 2020; Hou et al. 2017; Nishioka et al. 2006; Zhao et al. 2007). CD8^+^ T cells, known for their cytotoxic function in clearing infection and targeting tumor cells, were shown by a seminal work to have a regulatory subset that dampens the immune response by suppressing CD4^+^ T cells (Cantor et al. 1978; Eardley et al. 1978). Recently, it has been demonstrated that CD8^+^ regulatory T cells (Tregs) target active CD4^+^ T cells in an antigen‐specific manner (Saligrama et al. 2019). A series of investigations established the identity of CD8^+^ Tregs as the subset of CD8^+^ T cells coexpressing memory T‐cell markers CD44 and CD122 as well as natural killer (NK) cell marker Ly49 in unmanipulated mice (Kim et al. 2010, 2011; Rifa'i et al. 2004). Importantly, these CD8^+^ Tregs do not express Foxp3, instead, their regulatory function relies on the transcription factor Helios (encoded by *Ikzf2*) (Kim et al. 2015). We have described a unique molecular mechanism controlling the homeostasis and function of CD8^+^ Tregs involving TGF‐b (transforming growth factor‐b) and transcription factor Eomesodermin (Eomes) (Mishra et al. 2021). Furthermore, we provided a notable example that CD8^+^ Tregs control the development of spontaneous germinal center reactions and autoimmune diseases independent of CD4^+^ Tregs (Mishra et al. 2021). Despite its nonredundant role in controlling autoimmunity (Kim et al. 2010; Li et al. 2022; Mishra et al. 2021), there is a notable gap in our understanding of how aging affects CD8^+^ Tregs. Hence, we aimed to explore the age‐dependent changes in CD8^+^ Tregs.

Thymic involution, a hallmark of the aging immune system, leads to a reduction in naive T cell generation, which drives the homeostatic proliferation of remaining T cells (Goronzy and Weyand 2019; Miller 2020; Mittelbrunn and Kroemer 2021). Recent studies have identified a subset of CD8^+^ T cells, termed “virtual memory T cells” (T_VM_), in mice (Lee et al. 2013) and humans (White et al. 2016) which despite being antigen naive, expresses memory phenotype due to homeostatic proliferation and accumulate with age. When investigating CD8^+^ Tregs, we noticed that wild‐type (WT) CD8^+^ Tregs were CD122^hi^ and CD49d^low^, which are shared markers defining T_VM_ cells. Besides, both T_VM_ and CD8^+^ Tregs rely on Eomes, a transcription factor known to promote the homeostasis of T_VM_ (White et al. 2016) and the homeostasis/localization of CD8^+^ Tregs (Mishra et al. 2021). The only surface marker to distinguish T_VM_ and CD8^+^ Tregs may be Ly49, which is not included in most previous T_VM_ research. Thus, a large portion of bulk T_VM_ investigations in mice is likely confounded by the contamination of CD8^+^ Tregs and there is no report to provide a side‐by‐side comparison between T_VM_ and CD8^+^ Tregs.

In this study, we first established a clear distinction between T_VM_ and CD8^+^ Tregs. Then, we demonstrated that the population of CD8^+^ Tregs exhibited bi‐phasic dynamics during aging, that is, the first phase of age‐dependent accumulation until around 12 months followed by the second phase of age‐dependent decline. This unique bi‐phasic pattern is distinct from other T cell subsets, including CD4^+^ Tregs and T_VM_. Moreover, aging CD8^+^ Tregs were found to show transcriptional and phenotypic characteristics of NK cells, this included enrichment of NK cell receptor genes more importantly *Klrk1* that encodes NKG2D, which suppresses CD8^+^ Tregs in middle‐aged, but not young mice. Interestingly, no apparent functional defects were identified in aged CD8^+^ Tregs. Age‐dependent decline of a similar CD8^+^ Treg subset was confirmed in adult humans. Together, we establish age‐dependent bi‐phasic population dynamics for CD8^+^ Tregs in both mice and humans.

## 2 Results

### 2.1 CD8 ^+^ Tregs and T_VM_ are Functionally Distinct and Controlled by Different Molecular Mechanisms

**FIGURE 1:**
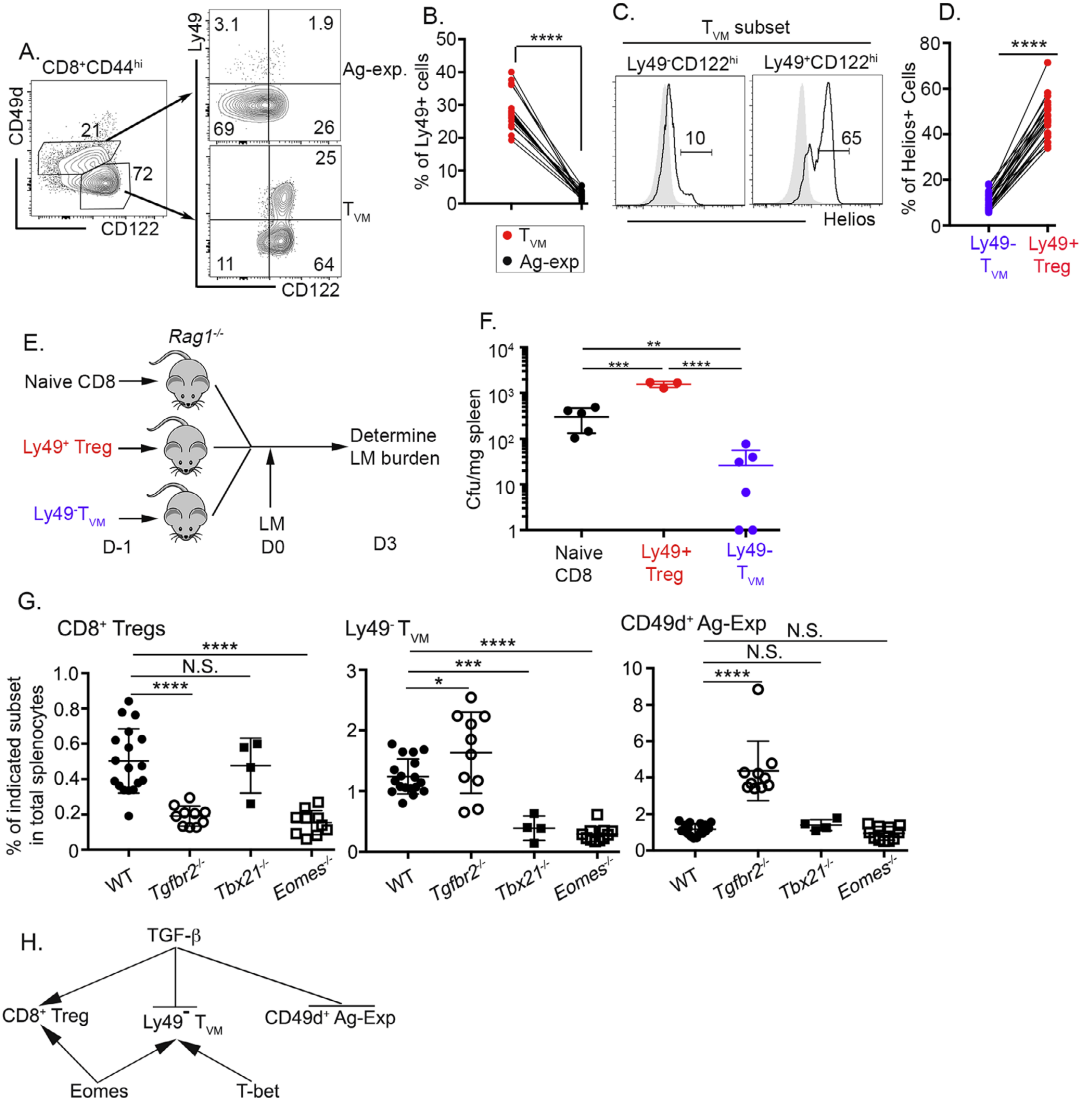
CD8^+^ Tregs and T_VM_ are functionally and molecularly distinct. (A) Representative FACS profiles of pregated CD8^+^CD44^hi^ T cells isolated from WT spleen (4–5 months). (B) The percentage of Ly49^+^ cells in CD49d^+^ antigen‐experienced CD8 versus CD49d^−^CD122^hi^ T_VM_. (C) Helios expression in Ly49^−^CD122^hi^ and Ly49^+^CD122^hi^ subsets within CD49d^−^CD122^hi^ T_VM_ gate. Gray represents naive CD8^+^ T cells to serve as a negative staining control. (D) The percentage of Helios^+^ cells (statistical analysis for C). Each pair of connected symbols in (B) and (D) represents the results from an individual mouse. (E) Schematics for (F). Naive, Ly49^+^CD8^+^ Tregs, and Ly49^−^T_VM_ were FACS sorted from WT spleen, and a same number of sorted cells were adoptively transferred into *Rag1* ^*−/−*^ mice followed by intravenous LM infection. LM bacterial burden in the spleen was measured 3 days later (shown in F). (G) The percentage of CD8^+^ Tregs (CD44^hi^CD122^hi^Ly49^+^CD8^+^, left), Ly49^−^T_VM_ (CD44^hi^CD122^hi^CD49d^−^Ly49^−^CD8^+^, middle), and CD49d^+^ antigen‐experienced CD8 (CD44^hi^CD49d^+^CD8^+^, right) in total splenocytes isolated from naive 3‐ to 6‐month‐old mice. (H) Differential control of CD8^+^ Tregs and Ly49^−^T_VM_. Each symbol in (F) and (G) represents the results of an individual mouse. Pooled results from two (F) or more than four (B, D, and G) independent experiments are shown. Statistical significance of data presented (means ± SEM) was determined using paired Student's *t*‐test or one‐way ANOVA (N.S. not significant; *p < 0.05; **p < 0.01; ***p < 0.001; and ****p < 0.0001).

In most mouse T_VM_ publications, bulk T_VM_ is generally defined as CD44^hi^CD122^hi^CD49d^−^ CD8^+^ T cells in uninfected and unimmunized mice (i.e., naive mice) (Haluszczak et al. 2009; Lee et al. 2013). In the CD8^+^ Treg field, CD8^+^ Treg is often described as CD44^hi^CD122^hi^Ly49^+^ CD8^+^ T cells (Kim et al. 2011). Our previous research has shown that most CD8^+^ Tregs in WT naive mice are CD49d^−^ (Mishra et al. 2021), identical to the commonly accepted T_VM_ definition. Since T_VM_ provide both antigen‐specific and bystander immune protection while CD8^+^ Tregs are immune regulatory, we would like to provide a clear distinction between these two phenotypically similar CD8^+^ T cell subsets to avoid potential confounders. As shown in Figure 1A,B, we could easily separate CD44^hi^CD8^+^ T cells into T_VM_ (CD49d^−^CD122^hi^) and antigen‐experienced (CD49d^+^) subsets in WT naive mice (4‐5‐month). In contrast to the CD49d^+^ subset, T_VM_ contained a distinct Ly49^+^ population representing CD8^+^ Treg (Figure 1A,B). Helios is a transcription factor required for the suppressive capacity of CD8^+^ Tregs (Kim et al. 2015). Within the T_VM_ gate, the Ly49^+^ subset was highly enriched for Helios^+^ cells (Figure 1C,D). To further elucidate the functional distinction, Ly49^+^CD8^+^ Tregs and Ly49^−^T_VM_ were FACS sorted from WT spleen and adoptively transferred into immunodeficient *Rag1* ^*−/−*^ mice together with a naive CD8^+^ T cell control group (Figure 1E). All *Rag1* ^*−/−*^ recipient mice were infected by *Listeria monocytogenes* . Three days later, the bacterial burden was measured. As expected, T_VM_ provided immediate immune protection likely via bystander memory T cell activation. In stark contrast, Ly49^+^CD8^+^ Treg did not provide any detectable protection (Figure 1F). Together, these results demonstrate that the commonly used T_VM_ gate is heavily contaminated by CD8^+^ Tregs. Including Ly49 is essential to distinguish bona fide T_VM_ (Ly49^−^) from CD8^+^ Tregs.

We have discovered that TGF‐b and Eomes synergistically promote CD8^+^ Tregs while Eomes‐related transcription factor T‐bet (encoded by *Tbx21*) is not apparently involved in CD8^+^ Tregs (Mishra et al. 2021). We examined CD8^+^ Tregs, Ly49^−^T_VM_ and CD49d^+^ antigen‐experienced CD8^+^ T cells in various mature T cell–specific conditional knockout mice mediated by distal *Lck*‐Cre, that is, *Tgfbr2* ^f/f^d*Lck*‐Cre (simplified as *Tgfbr2* ^*−/−*^), *Eomes* ^f/f^d*Lck*‐Cre (*Eomes* ^*−/−*^), and *Tbx21* ^*f/f*^d*Lck*‐Cre (*Tbx21* ^*−/−*^). While Eomes was similarly required for both CD8^+^ Treg and Ly49^−^T_VM_, T‐bet was only required for Ly49^−^T_VM_, and TGF‐b played opposite roles in CD8^+^ Treg versus Ly49^−^T_VM_ (Figure 1G). In CD49d^+^ antigen‐experienced T cells, only TGF‐b was actively involved (Figure 1G). As summarized in Figure 1H, CD8^+^ Treg and Ly49^−^T_VM_ are controlled by distinct molecular machinery.

Although TGF‐b is required for the homeostasis of CD8^+^ Tregs in vivo, during in vitro CD8^+^ T cell activation, the addition of TGF‐b was not sufficient to induce the expression of Ly49. However, TGF‐b robustly boosted Helios expression under the same setting, consistent with our previous finding (Mishra et al. 2021) (Figure S1).

Together, our results demonstrate that T_VM_ and CD8^+^ Treg exhibit distinct functions and are controlled by different underlying molecular mechanisms. Including Ly49 staining to distinguish CD8^+^ Treg from T_VM_ is critical to separate these two phenotypically overlapping subsets and avoid confounders.

### 2.2 Age‐Associated Changes in the CD8 ^+^ CD44^hi^CD122^hi^Ly49 ^+^ Tregs

**FIGURE 2:**
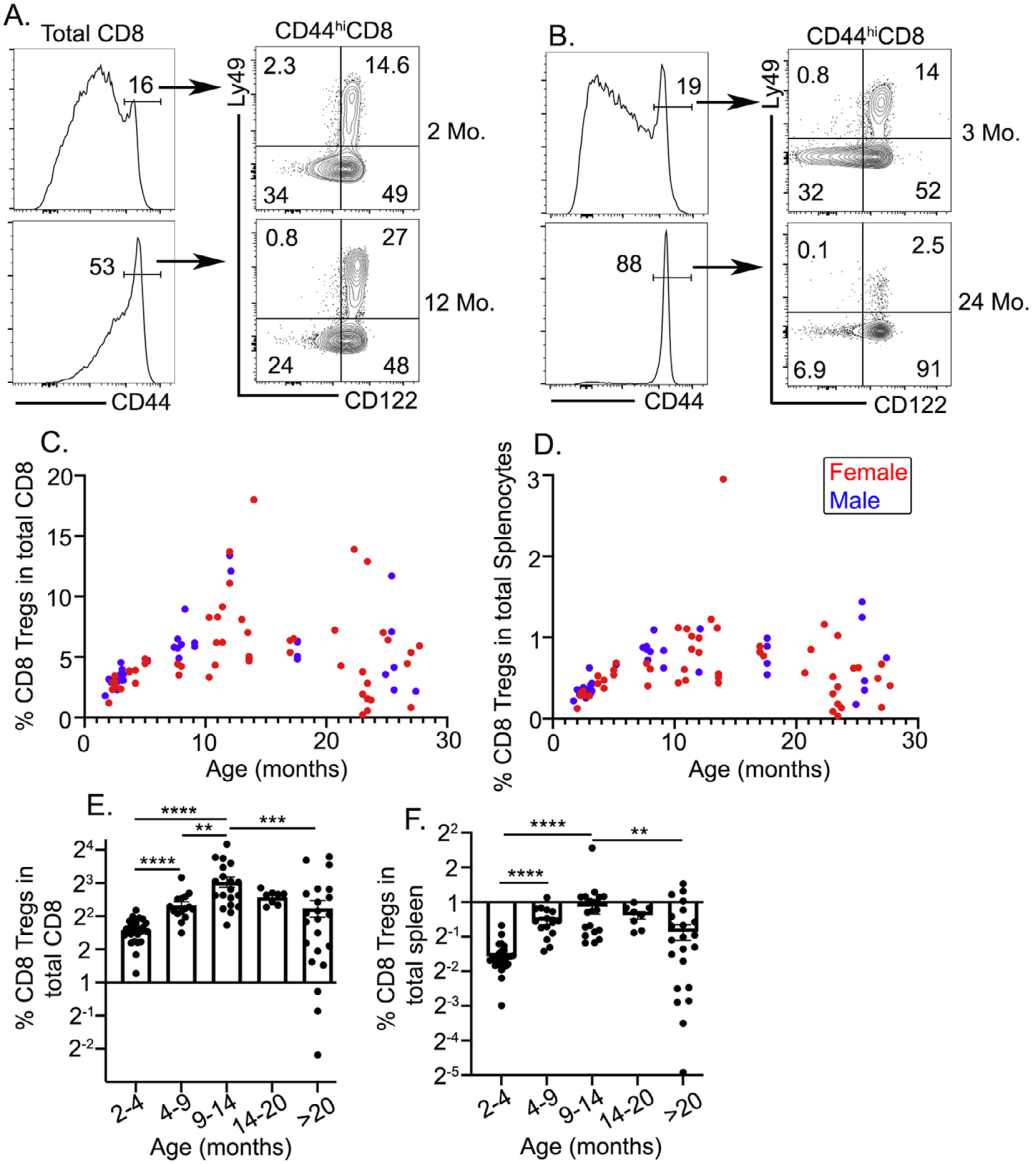
Age‐dependent bi‐phasic changes in CD8^+^ Tregs in mice. Splenocytes from mice of various age groups were harvested and stained for CD8^+^ Tregs and were analyzed using flow cytometry. Gating strategy for CD122^+^Ly49^+^CD44^+^CD8^+^ T cells (CD8^+^ Tregs) in (A) young (2 months old) and middle‐aged mice (12 months old) and (B) young (3 months old) and old aged mice (24 months old). (C) Scatter plot of CD8^+^ Tregs as a percentage of total CD8^+^ cells across a 2‐ to 30‐month age range, with individual data points for male (blue) and female (red) mice. (D) Scatter plot showing the percentage of CD8^+^ Tregs in the total spleen cell population by age and sex, with male (blue) and female (red) mice compared. Percentage of CD8^+^ Tregs among (E) the total CD8^+^ population and (F) total splenocytes at different age intervals, indicating a peak in middle‐aged mice followed by a decrease in older mice. Each symbol represents the results of an individual mouse. Statistical significance of data presented (means ± SEM) was determined using one‐way ANOVA or Student's *t*‐test (N.S. not significant; *p < 0.05; **p < 0.01; ***p < 0.001; and ****p < 0.0001).

Next, to elucidate the age‐dependent changes in CD8^+^ Tregs, we analyzed their frequency in young (2–3 months), middle‐aged (9–14 months), and old (> 20 months) mice. Consistent with previous studies (Decman et al. 2012), we observed an age‐dependent decline in the frequency of naive CD8^+^ T cells (CD8^+^CD44^−^) (Figure 2A,B), whereas memory‐like CD8^+^ T cells (CD8^+^CD44^hi^) increased significantly with age (Figures 2A,B and S2B). Subsequent analysis of these CD44^hi^CD8^+^ T cells for CD122 and Ly49 expression showed a significantly increased proportion of CD122^high^Ly49^+^ (CD8^+^ Tregs) in middle‐aged mice compared to young mice (Figure 2A,C–F). However, CD8^+^ Tregs substantially decreased in older mice (> 20 months) (Figure 2B–F). The overall percentage of CD8^+^ Treg within the total CD8^+^ T cell population initially increased, reaching a maximum in the middle‐aged mice, before declining with advancing age (Figure 2C,E). Also, we saw a similar pattern in total splenocytes (Figure 2D,F). Notably, we did not see any significant sex‐based differences (Figure 2C,D). Consistent with the frequency of CD8^+^ Tregs, the absolute number of CD8^+^ Tregs in total splenocytes also followed a similar age‐dependent pattern (Figure S2A). This confirms that selective dynamics of CD8^+^ Tregs does not change with splenocyte number.

As we have discussed before (Figure 1), CD8^+^ Tregs are often included in T_VM_ gate in the absence of Ly49 staining. Here, we revisited the population change in T_VM_ during aging. We showed that the frequency of Ly49^−^ T_VM_ increases with age (Figure S2B), similar to previous findings (Quinn et al. 2018), and no bi‐phasic pattern was observed, further demonstrating the importance of Ly49 in distinguishing T_VM_ and CD8^+^ Tregs. Thus, CD8^+^ Tregs, but not T_VM_ or total CD44^hi^CD8^+^ T cells, exhibit this unique age‐dependent pattern. In the CD4^+^ T cell population, CD4^+^PD1^+^CXCR5^+^ follicular helper T cells (T_FH_) and CD4^+^Foxp3^+^ (CD4^+^ Treg) showed an age‐related increase (Figure S3A–C). Spontaneous germinal center B cells remain stable during aging (Figure S3D). Therefore, unlike the other immune cell populations, CD8^+^ Treg has a distinct kinetics pattern with aging.

### 2.3 Age‐Dependent Alterations in Helios

**FIGURE 3:**
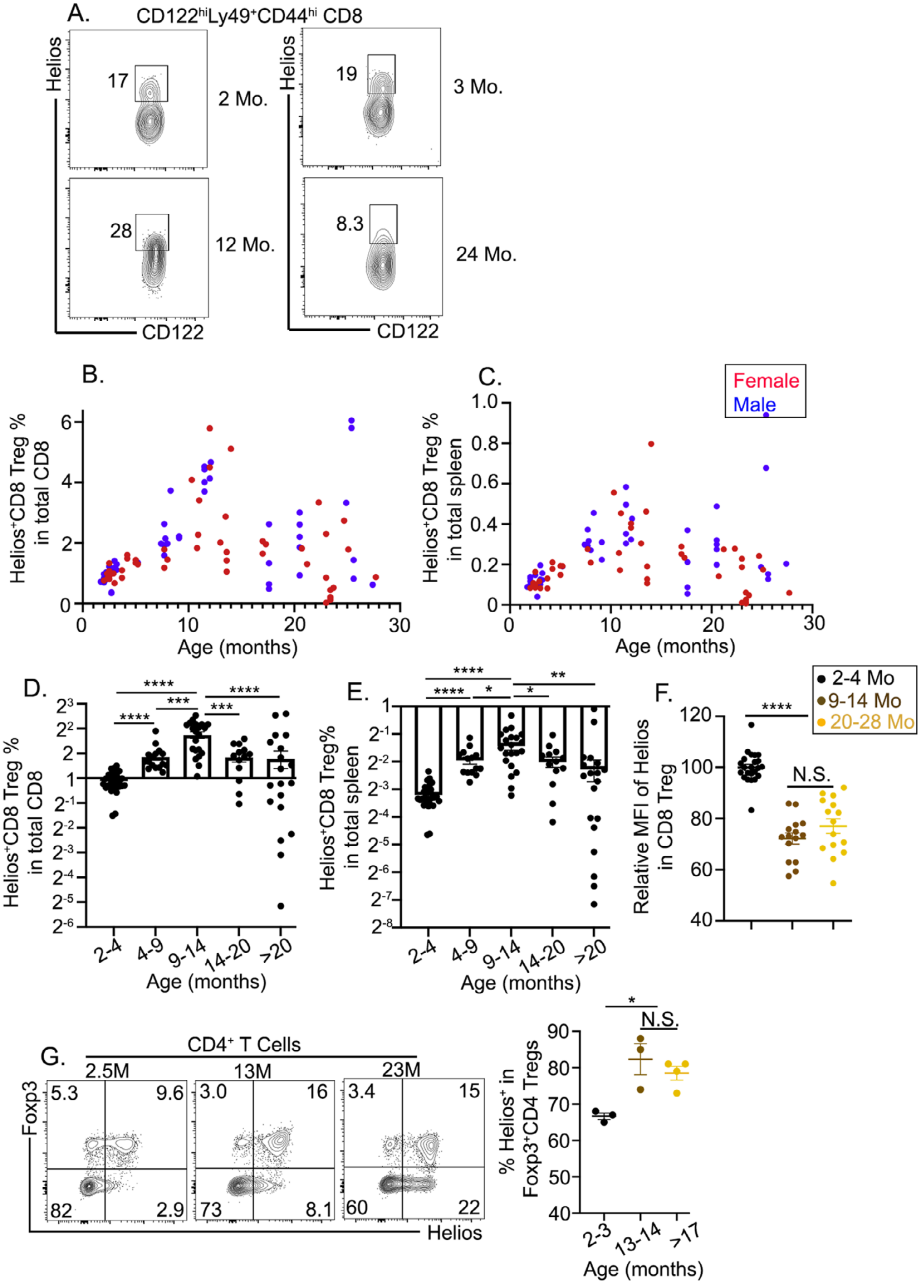
Age‐dependent bi‐phasic changes in Helios^+^CD8 Tregs in mice. Similar setup to Figure 2. Gating strategy for Helios^+^CD8^+^ Tregs in (A) young (2–3 months old) and middle‐aged mice (12 months old) and old mice (24 months old). (B) Scatter plot of Helios^+^CD8^+^ Tregs as a percentage of total CD8^+^ cells across a 2‐ to 30‐month age range, with individual data points for male (blue) and female (red) mice. (C) Scatter plot showing the percentage of Helios^+^CD8^+^ Tregs in the total spleen cell population by age and sex, with male (blue) and female (red) mice compared. Percentage of Helios^+^CD8^+^ Tregs among (D) the total CD8^+^ population and (E) total splenocytes at different age intervals, indicating a peak in middle‐aged mice followed by a decrease in older mice. (F) Relative mean fluorescence intensity (MFI) of Helios expression in pregated Helios^+^CD8^+^ Tregs across age groups. (G) Representative FACS profiles of pregated splenic CD4^+^ T cells are shown on the left. The percentage of Helios^+^ cells in pregated Foxp3^+^CD4^+^ T cells is shown on the right. Each circle represents the results from an individual animal. The statistical significance of data presented (means ± SEM) was determined using one‐way ANOVA or Student's *t*‐test (N.S., not significant; *p < 0.05; **p < 0.01; ***p < 0.001; and ****p < 0.0001).

Helios, a transcription factor essential for the regulatory function of CD4^+^ Foxp3^+^ Tregs and CD8^+^ Tregs (Kim et al. 2015), was assessed for age‐related expression changes. When first gated on CD122^hi^Ly49^+^ CD8^+^ Tregs, the frequency of Helios^+^ cells was highest among middle‐aged CD8^+^ Tregs compared with their younger and older counterparts (Figure 3A). Helios^+^CD8^+^ Treg frequency within total CD8^+^ T cells (Figure 3B,D) or within total splenocytes (Figure 3C,E) exhibited an identical bi‐phasic pattern and no sex‐related difference was detected (Figure 3B,C). When pregated on Helios^+^CD8^+^ Tregs, Helios expression level was found to be higher in young cells than in those from middle‐aged and old mice on a per‐cell basis (Figure 3F). In contrast, Helios expression was increased in Foxp3^+^ CD4 Tregs during aging (Figure 3G). Taken together, Helios^+^CD8^+^ Treg population size follows the same age‐dependent bi‐phasic pattern while young Helios^+^CD8^+^ Tregs carry the highest level of Helios expression on a per‐cell basis.

### 2.4 Expression Profiles and Age‐Related Dynamics of Ly49 Receptors in CD8 ^+^ CD44^hi^CD122^hi^ Tregs

Ly49 receptors, a family of Type II membrane glycoproteins, are traditionally recognized for their role in regulating NK cell activity (Rahim et al. 2014). In CD8^+^ T cells, CD8^+^ Treg suppressive activity is almost exclusively limited to Ly49‐expressing CD8^+^CD122^+^ T cells (Kim et al. 2011). Because in CD8^+^ Treg research, the commonly used monoclonal antibody for Ly49 (clone 14B11) recognizes Ly49C, Ly49F, Ly49I, and Ly49H, we sought to characterize the expression of individual Ly49 receptors and their dynamics across different age groups. We would like to determine whether the bi‐phasic pattern is limited to one Ly49 isoform‐defined CD8^+^ Tregs or can be extended to other Ly49 isoforms.

**FIGURE 4:**
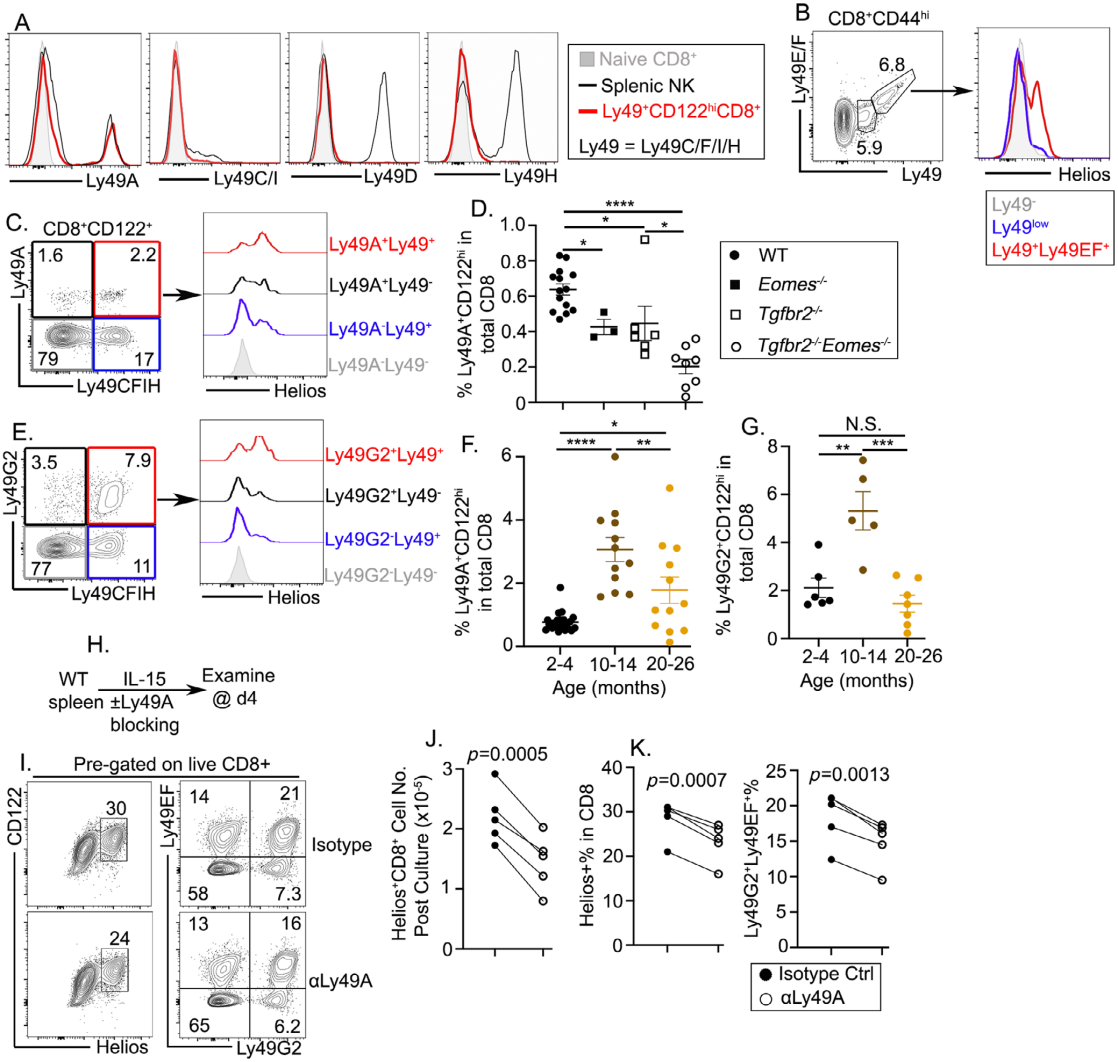
Expression profiles of Ly49 isoforms in CD8^+^ Tregs and their age‐related dynamics. (A) The expression of Ly49A, Ly49C/I, Ly49D, and Ly49H on naive CD8^+^ T cells, splenic NK cells, and Ly49^+^CD122^+^CD8^+^ Tregs. (B) Gating strategy of memory‐like CD8^+^ T cells (left) and the expression of Helios on different subsets of Ly49‐expressing CD8^+^CD44^high^ T cells isolated from WT spleen. (C) Gating strategy of Ly49A and Ly49C/F/I/H on CD8^+^CD122^+^ T cells (left) and the expression of Helios on different subsets of CD8^+^CD122^+^ T cells isolated from WT spleen. (D) Frequency of Ly49A^+^CD122^high^ cells among the CD8^+^ T cell population in WT, *Eomes* ^*−/−*^, *Tgfbr2* ^*−/−*^, and *Tgfbr2* ^*−/−*^ *Eomes* ^*−/−*^ mice. (E) Gating strategy of Ly49G2 and Ly49C/F/I/H on CD8^+^CD122^+^ T cells (left) and the expression of Helios on different subsets of CD8^+^CD122^+^ T cells isolated from the spleen of WT mice. Frequency of (F) Ly49A^+^CD122^high^ cells and (G) Ly49G2^+^CD122^high^ cells among the CD8^+^ T cell population across different age groups. (H) Experimental setup of in vitro Ly49A blocking assay. (I) Representative FACS profiles of pregated live CD8^+^ T cells after 4 days of culture. (J) Live Helios^+^CD8^+^ T cell number postculture. Equal numbers of CD8^+^ T cells from each mouse were cultured under two conditions, that is, with or without Ly49A blocking. Each pair of symbols represents the results from an individual mouse. (K) The percentage of Helios^+^ (left) and the percentage of Ly49G2^+^Ly49EF^+^ (right) among live CD8^+^ T cells after culture are shown. (D, F, G, J) Each symbol and (K) each pair of symbols represent the results of an individual animal. The statistical significance of the data presented (means ± SEM) was determined using one‐way ANOVA (*p < 0.05, **p < 0.01, ***p < 0.001, and ****p < 0.0001) for (D, F, G). Paired *t*‐test was used for (J, K).

We observed selective expression patterns of Ly49 receptors on CD8^+^ Tregs. Specific activating Ly49 receptors such as Ly49D and Ly49H and inhibitory receptors such as Ly49C/I were notably absent, while inhibitory receptor Ly49A was highly expressed (Figure 4A). The lack of expression of Ly49C/I/H suggested that 14B11 antibody recognized Ly49F on CD8^+^ Tregs, consistent with the diagonal staining pattern of Ly49 (14B11) versus Ly49E/F antibodies (Figure 4B). Importantly, Ly49F^+^ CD8^+^ Tregs were highly enriched for Helios^+^ cells (Figure 4B). This result is highly consistent with previous findings that Ly49F is the major isoform expressed by CD8^+^ Tregs (Kim et al. 2011; Li et al. 2022).

Ly49A expression did not completely overlap with Ly49F (14B11) staining. Instead, we could reliably detect single‐positive (i.e., Ly49A^+^Ly49F^−^ and Ly49A^−^Ly49F^+^) and double‐positive (Ly49^+^Ly49F^+^) cells when pregated on CD8^+^CD122^hi^ cells (Figure 4C). The heightened expression of Helios in cells coexpressing Ly49A and Ly49F suggested a potentially enhanced regulatory capacity of this subset (Figure 4C). We have recently demonstrated that Ly49F (14B11)‐defined CD8^+^ Treg population is controlled by TGF‐b and Eomes (Mishra et al. 2021). Indeed, Ly49A^+^CD122^hi^ CD8^+^ T cells were similarly dependent on both TGF‐b and Eomes as demonstrated by the defects in mature T cell–specific conditional KO mice for TGF‐bR, Eomes, and double‐conditional KO mice (Figure 4D). Taken together, these results strongly suggest that both Ly49A‐ and Ly49F‐defined CD8^+^ Tregs are controlled by similar molecular mechanisms. Age stratification revealed an almost identical bi‐phasic dynamic pattern in Ly49A^+^CD122^hi^CD8^+^ T cells, with a significant increase in middle‐aged mice (10–14 months) which then decreased in old mice (20–26 months) (Figure 4F).

Together with the previously mentioned isoforms, we examined the expression of Ly49G2 on CD122^hi^CD8^+^ T cells to further delineate the repertoire of Ly49 engagement. Notably, within the CD122^hi^CD8^+^ T cells, Ly49G2 single‐positive and Ly49G2/Ly49F double‐positive cells could be easily identified. Cells coexpressing Ly49G2 and Ly49F exhibited elevated levels of Helios (Figure 4E), suggesting its involvement alongside the other Ly49 receptors in shaping the immune regulatory functions of CD8^+^ Tregs. Furthermore, with age, we observed a similar profile‐like Ly49A^+^CD122^hi^ Tregs. The proportion of Ly49G2^+^CD122^hi^ Tregs increased significantly in middle‐aged mice (10–14 months), which then diminished in the old mice (> 20 months) (Figure 4G). Taken together, all Ly49 isoforms‐defined CD8^+^ Treg populations exhibit a similar bi‐phasic pattern with age.

Next, using an in vitro homeostatic culture system, we would like to determine the function of Ly49 receptors on CD8^+^ Tregs. IL‐15 signal is required for the homeostasis of CD8^+^ Tregs. Using Ly49A as an example, we would like to test the role of Ly49 receptors during the homeostasis of CD8^+^ Tregs (Figure 4H). Interestingly, even though Ly49A is expressed on a small subset of CD8^+^ Tregs, Ly49A blocking led to a subtle, but significant reduction in Helios^+^CD8^+^ Tregs (Figure 4I–K). Thus, continuous signals via Ly49 receptors are required for the maintenance of CD8^+^ Tregs. Individual Ly49 receptors are not redundant. Blocking a single Ly49 receptor is sufficient to impact CD8^+^ Tregs. The reduction in Ly49 expression in aged mice may contribute to the bi‐phasic pattern of CD8^+^ Treg population during aging.

### 2.5 Ly49^+^Helios^+^ CD8 ^+^ Tregs: Unique Noncytokine Producers During Homeostatic Proliferation

**FIGURE 5:**
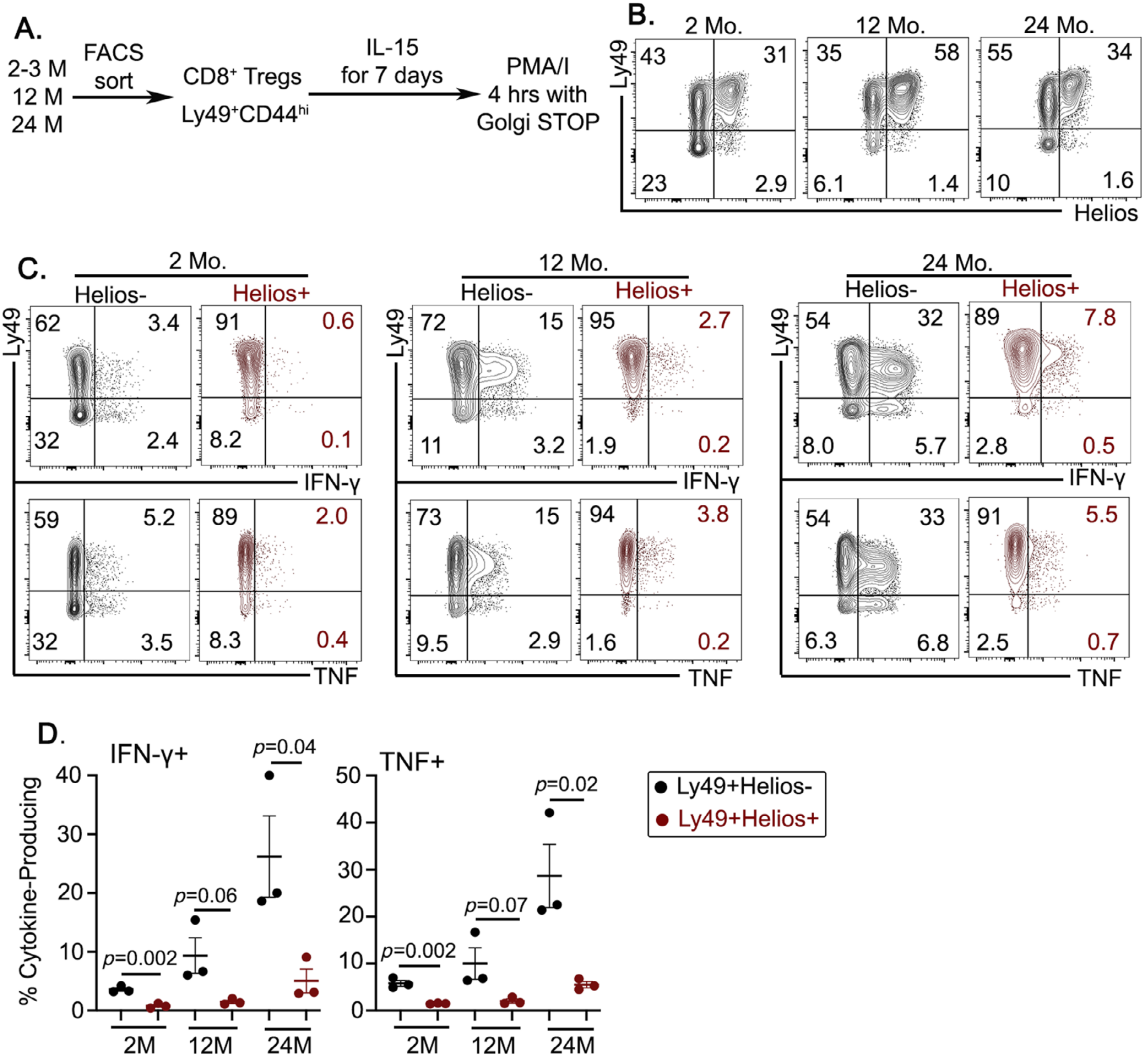
Helios^+^CD8^+^ Tregs are noncytokine producers during IL‐15 culture. (A) Schematic of the experiments. FACS‐sorted CD8^+^ Tregs (CD8^+^CD44^high^Ly49^+^) cells were cultured for 7 days in the presence of IL‐15 (100 ng/mL). Cells were then stimulated for 4 h by PMA/ionomycin in the presence of brefeldin A. (B) Representative FACS for Ly49^+^Helios^+^ T cells gated on CD8^+^CD122^+^. (C) Production of IFN‐γ (upper panel) and TNF‐α (lower panel) was then assessed by flow cytometry on Ly49^+^Hellios^−^ cells (black) and Ly49^+^Helios^+^ cells (ruby). Results are representative from three independent experiments with three mice per group. (D) Frequency of IFN‐γ^+^ (left) and TNF‐α^+^(right) cells within the indicated subset of different age groups. The statistical significance of the data presented (means ± SEM) was determined using Student's *t*‐test.

T_VM_ cells are known for IL‐15‐dependent expansion (Sosinowski et al. 2013). To determine whether there was any age‐dependent functional change in CD8^+^ Tregs during IL‐15‐dependent homeostatic proliferation, CD8^+^ Tregs were FACS sorted and cultured with IL‐15 for 7 days (Figure 5A). First, we noticed that IL‐15 could efficiently expand CD8^+^ Tregs, including Helios^+^CD8^+^ Tregs (Figure 5B). Next, a small subset of Helios^−^ CD8^+^ Tregs lost Ly49 expression during IL‐15 culture while Helios^+^CD8^+^ Tregs were phenotypically stable (Figure 5B). Subsequent stimulation with PMA/ionomycin on Day 7 revealed that Ly49^+^Helios^+^ Tregs could not produce cytokines during homeostatic proliferation, regardless of their age (Figure 5C,D). Intriguingly, cytokine production was confined to the Helios^−^ subset of CD8^+^ Tregs, and this response increased with age (Figure 5C,D). A similar trend was detected in freshly isolated CD8^+^ Tregs without IL‐15 culture. Ly49^+^Helios^−^ CD8^+^ Tregs could produce IFN‐g in an age‐dependent manner while Ly49^+^Helios^+^ CD8^+^ Tregs could not regardless of age (Figure S4A). This finding suggests that Ly49^+^Helios^−^ CD8^+^ T cells may not be fully committed to the Treg lineage and still carry effector/memory T cell features. In contrast, the Ly49^+^Helios^+^ subset of CD8^+^ Tregs does not engage in cytokine production and remains phenotypically stable during homeostatic expansion.

### 2.6 Ly49^+^Helios^+^ CD8 ^+^ Tregs: Unique Non‐Cytokine Producers During TCR Stimulation

**FIGURE 6:**
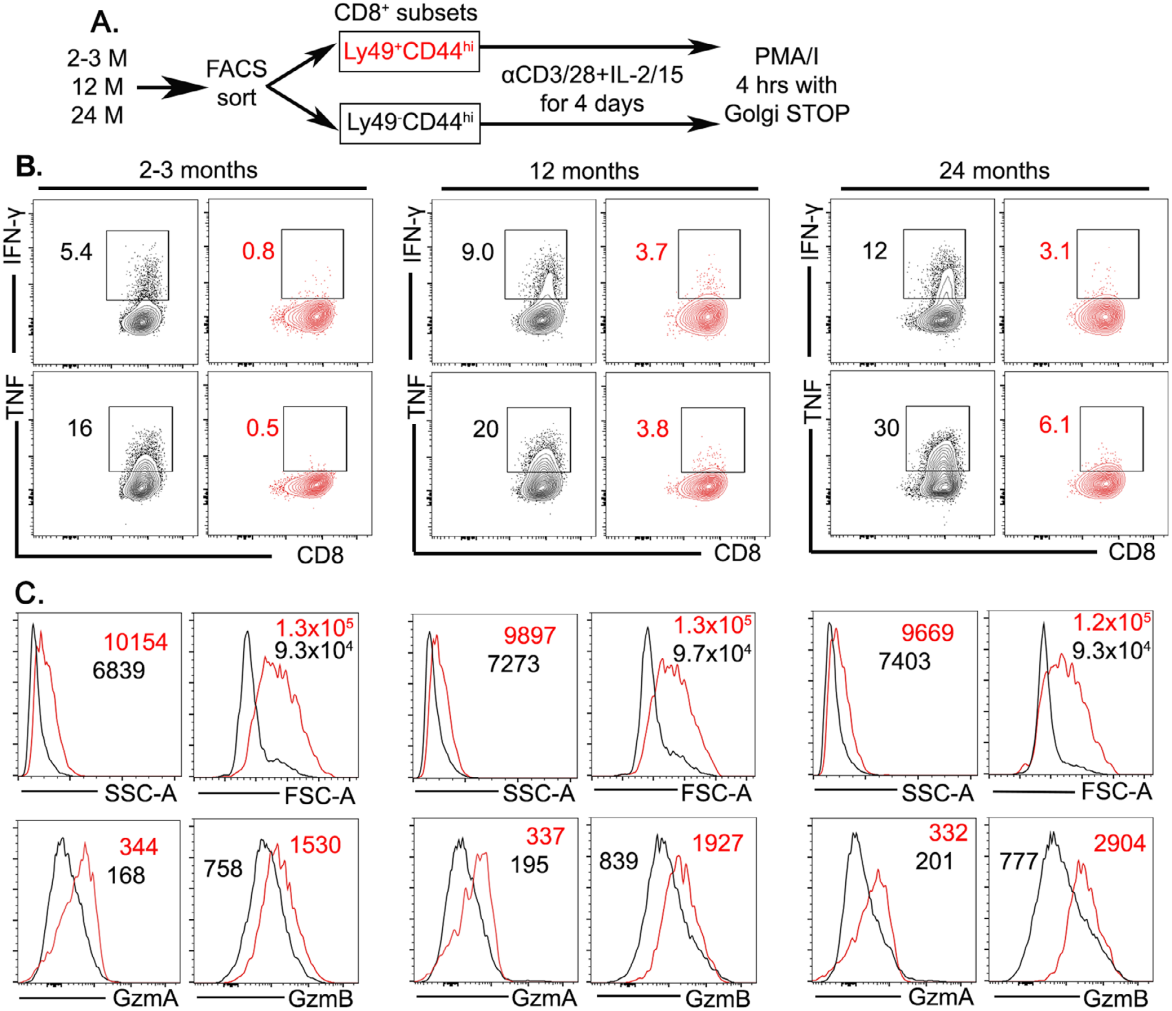
CD8^+^ Tregs upregulate the cytotoxic program upon TCR stimulation. (A) Schematic of the experiments. (B) Production of IFN‐γ (upper panel) and TNF‐α (lower panel) was assessed by flow cytometry on memory CD8^+^CD44^high^Ly49^−^ cells (black) and CD8^+^ Tregs (red). (C) Histograms of overlaid CD8^+^CD44^high^Ly49^−^ cells (black) and CD8^+^ Tregs (red) are shown. Numbers indicate MFI (mean fluorescence intensity) for color‐coded subsets. Results are representative from three independent experiments with three mice per group.

We next investigated the functional capacity of CD8^+^CD44^hi^CD122^hi^ Tregs during TCR stimulation. CD8^+^CD44^hi^CD122^hi^Ly49^+^ Tregs and CD8^+^CD44^hi^Ly49^−^ memory cells, sorted from different age groups, were activated using CD3/CD28 nanobeads along with IL‐2 and IL‐15 for 4 days. On the 4th day, cells were restimulated with PMA/ionomycin for 4 h to induce cytokine production (Figure 6A). Following the restimulation, memory cells (sorted CD8^+^CD44^hi^Ly49^−^ T cells) produced TNFα and IFNγ, more so in older cells compared to younger and middle‐aged groups (Figure 6B). In contrast to memory T cells, CD8^+^ Tregs, irrespective of age, did not produce cytokines in response to TCR stimulation (Figure 6B), highlighting a significant functional contrast with memory T cells. Furthermore, no IL‐10 production was detected in freshly isolated CD8^+^ Tregs during TCR stimulation (Figure S4B). In contrast to cytokine production, CD8^+^ Tregs robustly upregulate cytotoxic programs during TCR stimulation. Compared with Ly49^−^ memory CD8^+^ T cells isolated from the same animal, both granzyme A and granzyme B were substantially elevated in CD8^+^ Tregs regardless of age (Figure 6C). Thus, CD8^+^ Tregs are likely derived from a unique differentiation path with lost cytokine production but elevated cytotoxic capacity.

### 2.7 Apparently Normal Regulatory Function of Aged CD8 ^+^ Tregs

To determine the function of aged CD8^+^ Tregs in vivo, we took advantage of our recently established *Tgfbr2* ^*f/f*^ *Eomes* ^*f*/f^dLck‐Cre mice (DKO) (Mishra et al. 2021). In these T cell–specific double‐conditional KO mice, defective CD8^+^ Treg compartment leads to a spontaneous germinal center (GC) reaction. Adoptive transfer of WT CD8^+^ Tregs into young DKO mice completely mitigates the GC response (Mishra et al. 2021). CD8^+^ Tregs isolated from 2‐month‐old and 12‐month‐old mice were separately transferred into 2‐month‐old DKO mice. Three months later, donor CD8^+^ Tregs and host GC response were examined (Figure S5A). Both 2 M and 12 M WT CD8^+^ Tregs could efficiently suppress the spontaneous GC response in DKO hosts (Figure S5B,C). Furthermore, the population size of donor CD8^+^ Tregs (Figure S5D) and the frequency of Helios^+^ cells (Figure S5E,F) were comparable between 2 M and 12 M donors.

To further validate these findings, 2 M, 12 M, and 24 M mice were infected by LCMV (lymphocytic choriomeningitis virus). Two weeks later, CD4 T_FH_ (CD4^+^CD44^hi^PD‐1^+^CXCR5^+^) cells and CD8^+^ Tregs were examined. The ratios of CD4 T_FH_ and CD8 Tregs were comparable between the three age groups. Together, we conclude that aged CD8^+^ Tregs are functional.

### 2.8 Aging‐Induced Transcriptomic Shifts in CD8 ^+^ Tregs: The Emergence of NK Cell–Like Features

**FIGURE 7:**
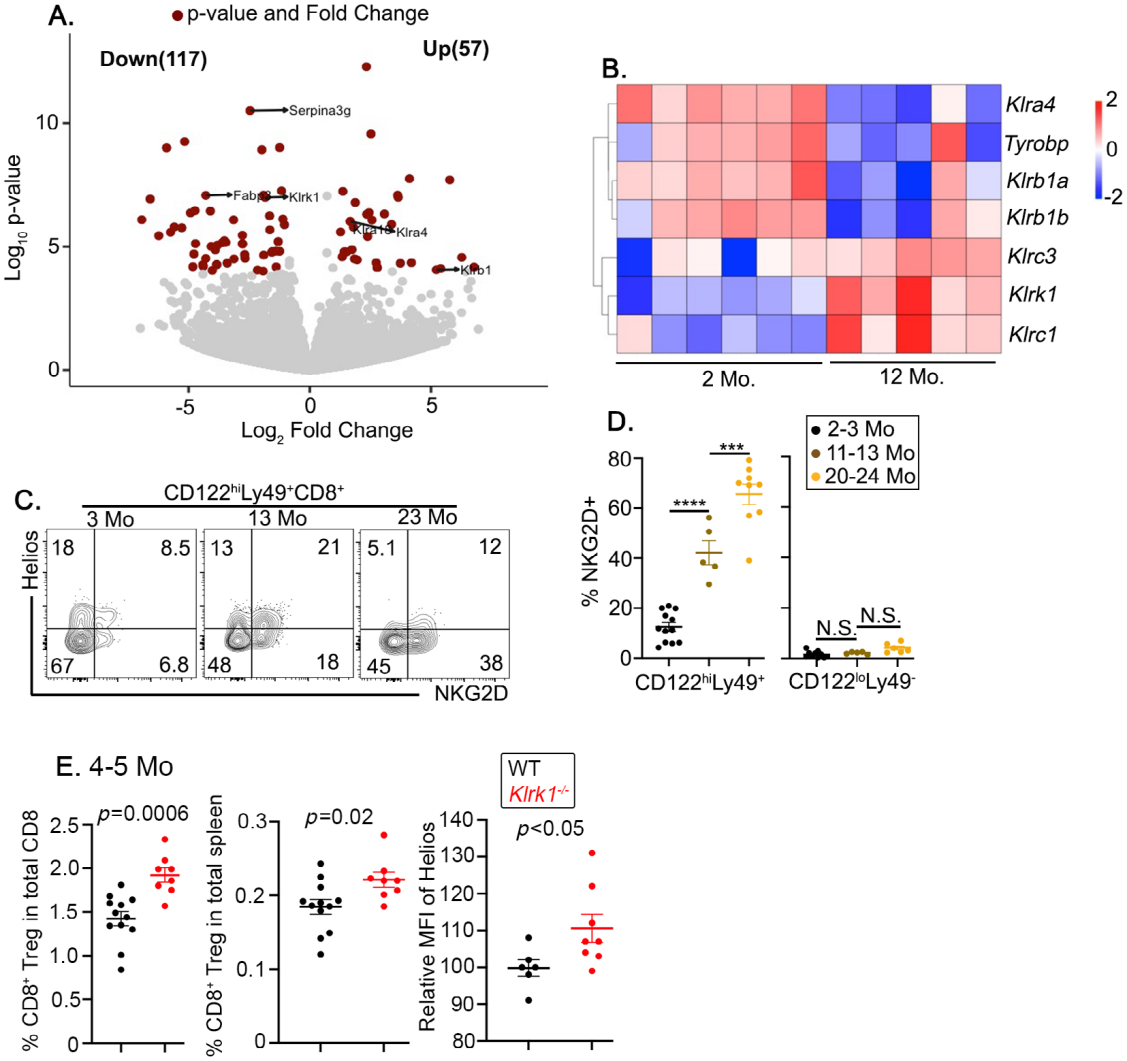
Transcriptional signatures of aging CD8^+^ Tregs. (A, B) CD8^+^ Tregs were FACS sorted from young (3 months) and Middle‐aged (12 months) mice, and their gene expression profiles were analyzed by RNA sequencing. (A) Volcano plot indicating differences in gene expression between young and middle‐aged CD8^+^ Tregs. (B) Heatmap of selected differentially expressed NK cell genes. Each column represents a biologically independent replicate. (C, D) Expression of NKG2D (encoded by *Klrk1*) on CD8^+^ Tregs from splenocytes of young (2–3 months), middle‐aged (11–13 months), and old‐aged (20–24 months) mice was analyzed by flow cytometry. (C) Representative FACS for the gating strategy of Helios^+^NKG2D^+^ T cells pregated on CD8^+^Tregs. (D) Frequency of NKG2D^+^ cells on CD122^high^Ly49^+^ (left) and CD122^high^Ly49^−^ (right). (E) Splenocytes of WT and *Klrk1* ^*−/−*^ mice 4–5 months were harvested and analyzed by flow cytometry. Frequency of CD8^+^ Tregs within total CD8^+^ T cells, total splenocytes, and relative MFI of Helios on CD8^+^ Tregs were shown. (D‐E) Each circle represents the results from an individual animal. The statistical significance of the data presented (means ± SEM) was determined using one‐way ANOVA or Student's t‐test (*p < 0.05, **p < 0.01, ***p < 0.001, and ****p < 0.0001).

To profile age‐dependent changes in CD8^+^ Tregs in an unbiased manner, we performed bulk RNA sequencing of FACS‐sorted CD8^+^ Tregs from young and middle‐aged mice. We found 174 differentially expressed genes (Figure 7A). Notably, middle‐aged CD8^+^ Tregs showed an upregulation of genes typically associated with NK cells, including *Fcgr2b* (which encodes the Fcγ receptor CD32) and NK‐receptor genes *Klrk1*, *Klrc3*, *Klrc2*, and *Klrc1* (Figure 7B,C). We next performed gene set enrichment analysis (GSEA), which showed that middle‐aged CD8^+^ Tregs were enriched for NK cell gene signature (Figure S6).

In addition to the upregulation of above‐mentioned key NK receptor genes, our findings indicated that middle‐aged CD8^+^ Tregs also downregulated several other NK cell receptors, including members of the killer cell lectin‐like receptor subfamily B (*Klrb1a*, *Klrb1b*) and subfamily A (*Klra4*), as shown in Figure 7B. *Klrb1a* and *Klrb1b*, which are orthologs of the human KLRB1 (CD161), are inhibitory receptors predominantly expressed in NK cells. Previous studies have suggested that human KIR^+^ CD8^+^ T cells exhibited lower levels of KLRB1 (Li et al. 2022). Consistently, RNA seq analysis of mouse CD8^+^ Tregs downregulated the *Klrb1* expression with age. Middle‐aged CD8^+^ Tregs also downregulated the transcripts of Ly49D, (encoded by *Klra4*), a NK cell–activating receptor. Thus, CD8^+^ Tregs appear to have a selective expression profile for NK receptors.

To confirm the upregulation of *Klrk1* transcripts, we assessed the NKG2D receptor (encoded by *Klrk1*) expression on CD8^+^ Tregs by flow cytometry. Consistent with transcriptomic data, NKG2D expression increased with age. Interestingly, the age‐dependent induction of NKG2D was more pronounced in Helios^+^ CD8^+^ Tregs than Helios^−^ CD8^+^ Tregs (Figure 7C). Importantly, the induction of NKG2D was not universal on all memory CD8^+^ T cells as we could not detect robust NKG2D expression on CD122^low^Ly49^−^CD44^hi^ CD8^+^ T cells (Figure 7D). Since NKG2D increased both at the transcript level and protein level, it led us to investigate whether NKG2D has functional implications in the aging CD8^+^ Tregs.

NKG2D is a Type II transmembrane receptor and is only expressed on murine CD8^+^ T cells upon activation, as opposed to its constitutive expression in humans (Raulet 2003). Since NKG2D promotes the function of memory CD8^+^ T cells (Chu et al. 2013), we would like to investigate the role of NKG2D on CD8^+^ Tregs. Since *Klrk1* ^*−/−*^ (*Klrk1* encodes NKG2D) mouse is known to develop tumors with age (Raju et al. 2016), we specifically used 4‐ to 5‐month‐old mice to avoid confounding factors associated with tumor progression. Significantly, we noted a higher occurrence of CD8^+^ Tregs in *Klrk1* ^*−/−*^ mice in comparison to age‐matched WT controls (Figure 7E left and middle). This supports our previous observation where CD8^+^ Tregs decreased in aged mice while they showed the highest NKG2D expression (Figures 2, 3, and 7C). In addition, CD8^+^ Tregs in *Klrk1* ^*−/−*^ mice showed higher expression of Helios in comparison to age‐matched WT CD8^+^ Tregs (Figure 7E right). When young mice (about 2 M) were examined, even though NKG2D expression was low on WT cells, we could detect a significant reduction in NKG2D level in *Klrk1* ^*−/−*^ mice (Figure S7A,B). However, no defects in CD8^+^ Treg population size and Helios expression were detected in young *Klrk1* ^*−/−*^ mice (Figure S7C,E), suggesting the impact of NKG2D on CD8 Treg is age dependent.

To test the function of NKG2D in CD8 Treg homeostasis, we performed in vitro NKG2D blocking experiments (Figure S7F). During IL‐15 culture, NKG2D blocking did not affect Helios^+^ CD8^+^ Tregs (Figure S7G). Together, this suggests that NKG2D might be involved in suppressing the homeostasis of CD8^+^ Tregs in an age‐dependent manner. However, the impact might not be direct, but rather via an altered cellular environment.

### 2.9 Age‐Related Dynamics and Molecular Characterization of a Treg‐Like CD8 ^+^ T Cell Subset in Human PBMCs

**FIGURE 8:**
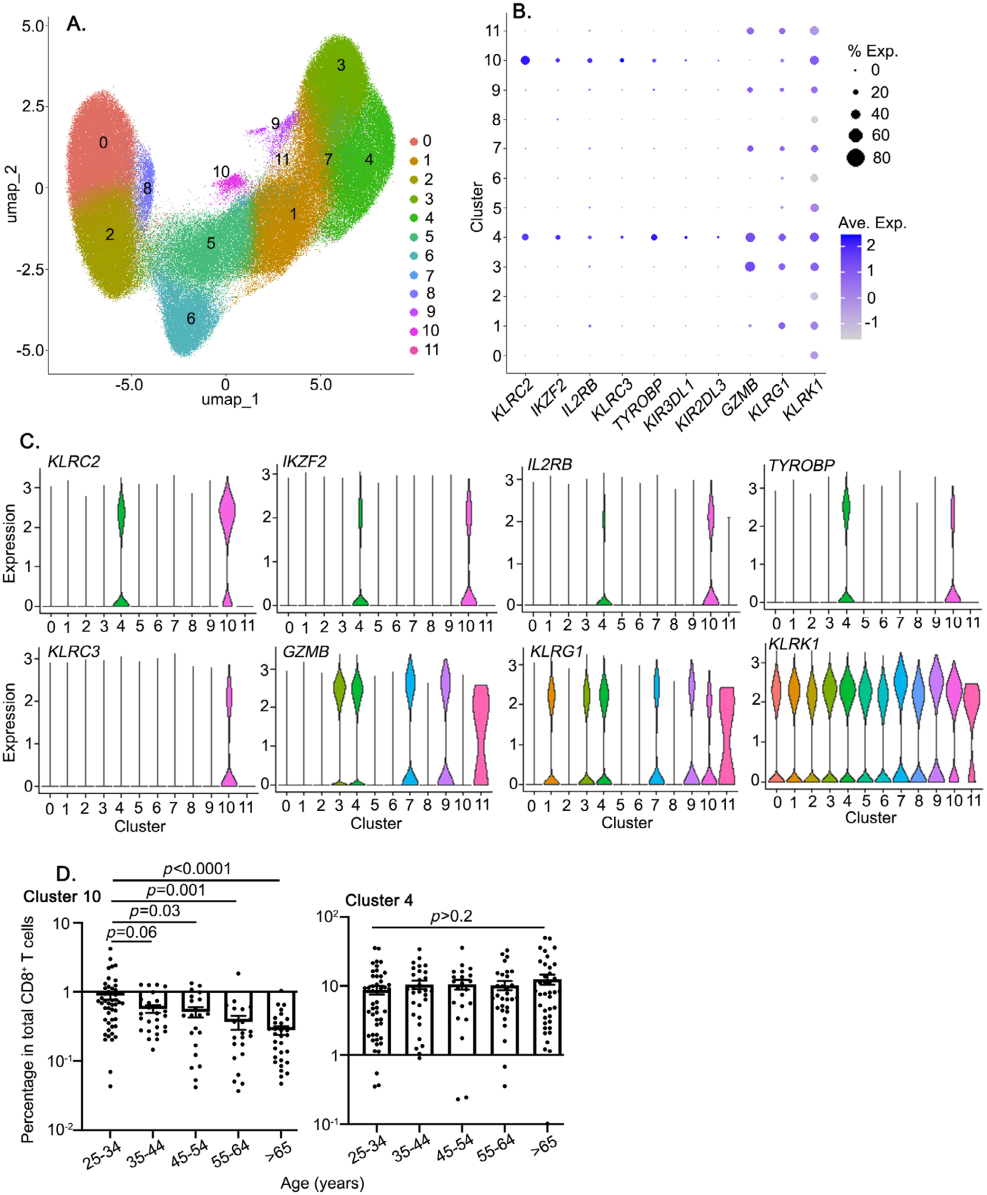
CD8^+^ Treg‐like cells in human PBMCs. scRNA‐seq dataset from PBMCs of healthy individuals aged 25–85 years old (Terekhova et al. 2023) was analyzed. (A) UMAP plot of CD8^+^ T cells colored by the cluster. (B) Dot plots for markers that characterize the CD8^+^ Treg‐like subset. (C) Violin plots showing normalized expression of KLRC2, IKZF2, KLRK1, IL2RB, KLRC3, KLRG1, GZMB, and TYROBP genes per cluster. (D) Bar plot showing the percentage of cells of Cluster 10 and Cluster 4 across age groups. Each dot represents an individual donor. The statistical significance of the data presented (means ± SEM) was determined using one‐way ANOVA.

To determine whether similar age‐related dynamics occur in human CD8^+^ T cells, we used a recently published single‐cell RNA‐sequencing dataset (Terekhova et al. 2023). This dataset is limited to healthy adults (*n* = 166) from 25 to 85 years old excluding all donors with inflammatory conditions or cancer and also excluded obese or smoking individuals. Unbiased clustering of CD8^+^ T cells from human peripheral blood mononuclear cells (PBMCs) defined 11 clusters. Of those 11 clusters, we found two subsets designated as Cluster 4 and Cluster 10 (Figure 8A) showed gene expression profile similar to mouse CD8^+^ Tregs, specifically Helios (*IKZF2*), CD122 (*IL2RB*), NKG2D (*KLRK1*), NKG2C (*KLRC2*), NKG2A (*KLRC3*), and human functional homolog of mouse Ly49 genes, KIRs (human killer cell immunoglobulin‐like receptors, e.g., *KIR3DL1* and *KIR2DL3*) (Figure 8B,C). On further examination, we found that Cluster 4 and Cluster 10 showed a difference in the expression of effector molecules, including granzyme B (*GZMB*) and *KLRG1* (Figure 8B,C). Cluster 4 showed high levels of *GZMB* and *KLRG1*, like other effector subsets, such as Clusters 3 and 9. Of note, Cluster 10 did not express *GZMB* and *KLRG1*. We have previously shown that WT mouse CD8^+^ Tregs do not express either granzymes or KLRG1 under steady states (Mishra et al. 2021). Taken together, Cluster 10 might be the human equivalent to CD8^+^CD44^hi^CD122^hi^Ly49^+^ Tregs identified in mouse models. To identify the age‐dependent changes of Cluster 10, we analyzed the distribution of Cluster 10 across various age groups and found a significant decline in its frequency with age (Figure 8D), from young adults at 25 years to the elderly over 65 years. This is similar to the observed age‐dependent reduction in the frequency of mouse CD8^+^ Tregs from middle age to older (Figures 2 and 3) and suggests a shared mechanism of CD8^+^ Treg attrition across species. In contrast, the effector molecule‐expressing Cluster 4 did not exhibit any age‐dependent changes. In summary, this strengthens that a CD8^+^ Treg‐like subset is present in human PBMCs and this population like its murine counterpart decreases with age in adult humans.

## 3 Discussion

Our results reveal the dynamics of CD8^+^ Treg throughout aging. Here, we found that in mice, there was an initial rise in Ly49^+^CD8^+^ Tregs until they reached around 10–12 months of age, after which there was a subsequent reduction (Figures 2 and 3). By using scRNA‐sequencing data, we also demonstrated that in humans, there was a steady decline in CD8^+^ Treg‐like cells from 25 to 85 years old (Figure 8C). However, this does not exactly match with the bi‐phasic dynamics of mouse Ly49^+^CD8^+^ Tregs. Considering the lifespan differences between mice and humans, where a 10‐month‐old mouse approximately corresponds to a 33‐year‐old human (Dutta and Sengupta 2016), our findings in the human scRNA‐seq dataset, particularly in the group of ages 25–35 years, might be akin to those in middle‐aged mice (10–12 months). This explains the consistent decrease in human Tregs with age. However, it would be particularly interesting to examine CD8^+^ Tregs in younger human age groups to fully understand their developmental trajectory in comparison to mice.

CD8^+^ Tregs and virtual memory cells (T_VM_) share a similar memory‐like phenotype and Eomes‐controlled transcription program, and are often grouped as a single population in most T_VM_ studies, identified as “CD8^+^CD44^hi^CD122^hi^CD49d^−^.” Here, our results have demonstrated that CD8^+^ Tregs and T_VM_ carry distinct functions and are controlled by different molecular mechanisms. Separating CD8^+^ Tregs and T_VM_ is essential to avoid confounding results. Our findings provide contrasting perspectives demonstrating that Ly49^+^Helios^+^ Tregs do not produce cytokines (including both inflammatory cytokines and regulatory cytokine IL‐10) in response to TCR stimulation (Figure 6). In contrast, CD8^+^ Tregs are skewed toward the cytotoxic lineage upon TCR stimulation, which is consistent with the requirement of perforin in CD8^+^ Treg suppression (Kim et al. 2011; Saligrama et al. 2019).

Anfossi et al. reported cytokine production by a fraction of Ly49^+^CD8^+^ T cells following IL‐15 treatment (Anfossi et al. 2004). Here, we showed that IL‐15‐mediated cytokine production solely in Ly49^+^Helios^−^ CD8^+^ T cells. Although Ly49^+^Helios^−^CD8^+^ T cells largely fall into the CD8^+^ Treg gate in most publications, the identity of Ly49^+^Helios^−^ CD8^+^ T cells remains to be determined. In other words, whether Ly49^+^Helios^−^ CD8^+^ T cells are true Tregs or effector/memory T cells is unknown. Notably, the CD8^+^Ly49^+^Helios^+^ subset, recognized for its suppressive capabilities (Kim et al. 2015), does not produce IFN‐γ and TNF‐α (Figure 6D,E). This further shows the functional divergence of CD8^+^ Tregs from T_VM_ and other effector/memory CD8^+^ T cells, highlighting their distinct roles in the immune response.

We demonstrate that aging CD8^+^ Tregs acquire several NK cell‐related gene signatures. Prior studies have demonstrated that chronic exposure to self‐antigen leads to upregulation of NKG2D in CD8^+^ T cells (Dhanji et al. 2004). Furthermore, recent work has indicated that Qa‐1‐restricted self‐antigen–specific CD8^+^ Tregs exhibit an age‐dependent increase in NKG2D expression, tracked up to 48 weeks of age (Kim et al. 2024). Our data are consistent with these findings, revealing that the Ly49^+^Helios^+^ subset of CD8^+^ T cells referentially upregulates NKG2D. Moreover, we observed a consistent increase in NKG2D expression up to 104 weeks of age.

NKG2D expression in CD8^+^ T cells is often linked with an effector phenotype and is considered a costimulatory molecule (Chu et al. 2013; Tsyklauri et al. 2023). In our study, CD8^+^ Tregs expanded in *Klrk1* ^*−/−*^ mice with higher expression of Helios in 4‐ to 5‐month‐old, but not in 2‐month‐old mice. Thus, in contrast to a costimulatory molecule for other effector/memory CD8^+^ T cell subsets, NKG2D may function as a coinhibitory molecule in CD8 Tregs in an age‐dependent manner. Considering the lack of impact of NKG2D blocking on purified CD8^+^ Tregs in vitro, it is possible that NKG2D exerts its effects on CD8^+^ Tregs via an altered microenvironment.

One interesting feature of aged CD8 Tregs is the greatly increased variation between individuals. As shown in Figure 2E,F and Figure 3D,E, in aged mice (> 20 months, compared with other groups), there is a much wider range of CD8 Treg population size. Thus, a large group of aged animals is required to accurately assess the function of CD8 Tregs. This feature of CD8 Treg prevents us from providing conclusive results measuring the in vivo function of aged CD8 Tregs, which represents a key limitation of the current work.

By using a publicly available scRNA‐seq dataset, we identified a murine CD8^+^ Treg‐like subset, “Cluster 10” in human PBMC. We found that Cluster 10 showed a gene expression profile similar to mouse Tregs, including *IL2RB*, *IKZF2*, and *KLRK1*. Interestingly, the frequency of Cluster 10 declined with age. While Cluster 10 shows the mouse Treg phenotype, Cluster 4 is characterized by higher levels of *KLRG1*, an effector CD8 marker, and granzyme B, indicative of cytotoxic capabilities. This is notable considering our previous findings that WT CD8^+^ Tregs do not produce granzyme B under homeostatic conditions and do not carry KLRG1 (Mishra et al. 2021). Cluster 10 also exhibited heterogeneous expression of KIR3DL1 and KIR2DL3, functional equivalents of murine Ly49 (Li et al. 2022; Rahim and Makrigiannis 2015). Further work is required to completely comprehend the unique functions of Clusters 10 and 4 in autoimmunity and aging, due to their complicated expression profile.

In summary, we performed a side‐by‐side comparison between CD8^+^ Tregs and T_VM_ to establish the necessity to distinguish these two subsets. We demonstrated the bi‐phasic dynamics of CD8^+^ Tregs during aging as well as highlighted their age‐associated functional identity, marked by an increased expression of NK cell‐related genes as well as altered Helios expression. Moreover, age‐related decline in “Cluster 10” subset, along with their unique molecular profiles, underscores the potential of CD8^+^ Tregs as promising targets for therapeutic strategies in age‐associated immune dysfunctions. However, further characterization should be done to dissect the CD8^+^ Treg‐specific molecular mechanisms in aging and autoimmunity.

## 4 Materials and Methods

### 4.1 Mice and Virus

C57BL/6J (B6) (Jax#000664) mice were originally obtained from the Jackson Laboratory. Young (2–4 months), middle‐aged (10–12 months) and old (20–28 months old) male and female mice were used. *Rag1* ^*−/−*^ mice (Jax#002216) were originally obtained from the Jackson Laboratory. Distal *Lck*‐Cre‐mediated T cell–specific conditional knockout mice for *Tgfbr2*, *Tbx21*, and *Eomes* (*Tgfbr2* ^*−/−*^ mice, *Tbx21* ^*−/−*^ and *Eomes* ^*−/−*^ mice) and double‐conditional KO mice (*Tgfbr2* ^*−/−*^ *Eomes* ^*−/−*^ mice) have been described before (Mishra et al. 2021). *Klrk1* ^*−/−*^ mice (Jax#022733) were purchased from Jackson Laboratory (Guerra et al. 2008). All mice were housed at our specific pathogen‐free animal facilities at the University of Texas Health San Antonio. All experiments were conducted in accordance with the National Institutes of Health Guide for the Care and Use of Laboratory Animals and were fully approved by the Institutional Animal Care and Use Committee of the University of Texas Health San Antonio. LCMV Armstrong infection was performed as described before (Wang et al. 2024). 2 × 10^5^ pfu LCMV Armstrong was injected into each mouse via an intraperitoneal route.

### 4.2 Staining for Flow Cytometry and Antibodies

Anti‐CD16/32 (2.4G2) was produced in the laboratory and used in all FACS staining as FcR blocker. Freshly isolated splenocytes of young, middle‐aged, and old aged mice were stained for surface antigens and incubated for 30 min at 4°C with the following fluorescence dye‐labeled antibodies specific for CD3 (17A2), B220 (RA3‐6B2), CD19 (1D3), CD4 (GK 1.5), CD44 (IM7), PD‐1 (J43), CXCR5 (SPRCL5), CD8β (H35‐17.2), Ly49C/F/I/H (14B11), CD122 (TM‐β1), CD25 (PC61.5), CD49d (R1‐2), Ly49D (4E5), Ly49I (YLi‐90), Ly49E/F (CM4), Ly49G2 (411), Ly49A (YE1), IL‐10 (JES5‐16E3), and NKG2D (CX5) were purchased from Thermo Fisher, Biolegend, and Cytek (Tonbo). For live cell staining, Ghost Dye Violet 510 (Cytek/Tonbo) was used. For intranuclear and intracellular staining, cells were fixed following surface staining and permeabilized with the FOXP3/transcription factor staining buffer kit (Cytek/Tonbo) for 45 min at room temperature and then stained with following antibodies Helios (22F6), Foxp3 (FJK‐16S), TNFa (MP6‐XT22), IFNg (XMG1.2), granzyme A (3G8.5) and granzyme B (GB11) at room temperature for 30 min. Washed and fixed samples were analyzed by BD FACSCelesta and FlowJo (TreeStar) software.

### 4.3 Lymphocyte Isolation and Cell Sorting

Murine spleen was harvested, homogenized, and red blood cells were lysed using ACK buffer (prepared in‐house). CD8^+^ T cells were isolated using Dynabeads mouse CD8^+^‐positive selection kit (Invitrogen). Isolated CD8^+^ T cells are then stained with surface antibodies CD8, CD44, Ly49, and CD122. CD8^+^CD44^+^Ly49^+^CD122^+^ cells (CD8^+^ Tregs) and CD8^+^CD44^+^Ly49^−^ cells (memory T cells) were purified by cell sorting using BD FACS Aria Fusion.

### 4.4 Listeria Infection in *Rag1* ^*−/−*^ Mice

Naive CD8^+^ (CD44^−^CD8^+^CD62L^+^), CD8^+^ Tregs (CD8^+^CD44^+^Ly49^+^CD122^+^), and T_VM_ (CD8^+^CD44^+^CD49d^−^CD122^+^Ly49^−^) were FACS sorted from the spleen of 4‐ to 6‐month‐old WT naive mice. 5 × 10^4^ sorted cells were adoptively transferred into each *Rag1* ^*−/−*^ recipient via tail vein. All recipients were infected by 2 × 10^3^ cfu *Listeria monocytogenes* intravenously as described before (Ma and Zhang 2015). Bacterial burden in the spleen was measured 3 days after infection.

### 4.5 In Vitro T Cell Stimulation and Intracellular Cytokine Staining

For TCR activation, FACS‐sorted CD8^+^ Tregs and memory T cells are cultured in vitro with Dynabeads Mouse T‐activator CD3/CD28 (Thermo Fisher) following the manufacturer's instructions. The culture was supplemented with IL2 (5 ng/mL, BioLegend) and IL15 (5 ng/mL, BioLegend).

For IL‐15 culture, FACS‐sorted CD8^+^ Tregs are cultured in vitro with IL15 (100 ng/mL) for 4 or 7 days.

For intracellular cytokine analysis, at the end of the culture period, cells were stimulated with 50 ng/mL PMA + 1 mg/mL Ionomycin (PMA and ionomycin are purchased from Sigma) for 4 h, and GolgiStop (BD) was also added. Cells were then stained with FACS antibodies for CD8, Ly49, Helios, TNFa, IFNg, granzyme A, and granzyme B as described above.

### 4.6 In Vitro Blocking Assays

For antibody‐blocking assays, 5‐ to 7‐month‐old WT CD8^+^ Tregs were cultured with IL15 (100 ng/mL). 10 mg/mL blocking antibodies were added throughout the culture. The following blocking antibodies were used: NKG2D (clone HMG2D, BioXcell) and Ly49A (clone YE1/48.10.6, BioLegend). At the end of the culture, live CD8^+^ Tregs were examined by FACS.

### 4.7 RNA‐Seq Analysis

Young and middle‐aged CD8^+^ Tregs were FACS sorted, and RNA was extracted from sorted cells using the Quick‐RNA MiniPrep according to the manufacturer's instructions (Zymo Research). RNA‐seq analysis was performed by Novogene. RNA‐seq data are accessible at GEO: GSE252718.

### 4.8 Analysis of Publicly Available scRNA‐Seq Data

We conducted analysis on a publicly available single‐cell RNA‐sequencing dataset of human PBMC from Terekhova (Terekhova et al. 2023) (syn49637038), specifically focusing on CD8^+^ T cells. The original authors had preprocessed this dataset to isolate CD8^+^ T cells, which we used for our analysis. We imported the dataset into R (v4.3.2) and utilized the Seurat package (version 5.0.1) for our analysis. We adapted the analysis workflow from the original author (Terekhova et al. 2023) with key steps including data normalization, variable feature identification with the exclusion of certain genes (TRA, TRB, IGH, IGK, and IGL), and data scaling. We applied PCA for dimensionality reduction, followed by Harmony integration for batch correction. Subsequently, we utilized UMAP for further dimensionality reduction and visualization. We also conducted clustering analysis using Seurat's FindNeighbors and FindClusters functions with a resolution of 0.8. We identified 12 clusters, among which Cluster 10 was of specific interest due to its distinctive characteristics within the CD8^+^ T cell population. We quantitatively analyzed the percentage of cells within Cluster 10 and Cluster 4 relative to the total CD8^+^ T cell count. The result was stratified by donor and across different age groups to explore age‐related differences in cluster composition. For visualization, we created Uniform Manifold Approximation and Projection (UMAP) plots. Furthermore, we used ggplot2v3.4.4 for creating dot plots and violin plots to visually represent gene expression data.

### 4.9 Statistics

Data were analyzed in Prism 10.

## Author Contributions

Conceptualization: S.S., S.M., N.M., G.H., M.R., C.M., and N.Z.; investigation: S.S., S.M., K.K.‐H.F., L.W., J.I., C.S., and C.M.; bioinformatics: S.S.; writing: S.S.; review and editing: S.S., N.M., G.H., M.R., C.M., and N.Z.; and funding acquisition: N.Z.

## Conflicts of Interest

The authors declare no conflicts of interest.

## Supporting information


**Figure S1.** TGF‐β induces Helios, but not Ly49 expression during in vitro CD8^+^ T cell activation. Purified naive OT‐1 TCR transgenic CD8^+^ T cells were activated by anti‐CD3/CD28 in the presence of 10μg/ml TGF‐β‐neutralizing antibody (clone#1D11.16.8, BioXcell) or 2.5ng/ml hTGF‐β1 (BioLegend). Two days later, live CD8^+^ T cells were FACS sorted and analyzed by bulk RNA‐seq. The mRNA levels for *Klra6* (A), *Klra7* (B), and *Ikzf2* (C) are shown. Each symbol represents the results from an independent culture sample. The *p*‐values were calculated by Student's *t*‐test.
**Figure S2.** Age distribution of CD44^hi^ and Ly49^+^ T_VM_ cells. (A) Number of CD8^+^ Tregs in the total splenocytes of young (2–3 months), middle‐aged (10–12 months), and old‐aged mice (20–24 months). The statistical significance of the data presented (means ± SEM) was determined using one‐way ANOVA (*p < 0.05, **p < 0.01). (B) Scatter plot of CD44^high^CD8^+^ (top) and Ly49^−^CD122^hi^CD49d^low^ T_VM_ cells (bottom) as a percentage of total CD8^+^ cells across a 2‐ to 28‐month age range, with individual data points for male (blue) and female (red). Each dot represents an individual mouse.
**Figure S3.** Age‐related kinetics of CD4^+^ Treg, CD4^+^T_FH_, and germinal center B cells. (A) Scatter plot illustrating the percentage of Foxp3^+^ cells within the total CD4^+^ cell population across a 0‐ to 27‐month age range. (B) Scatter plot showing the percentage of CD4^+^Foxp3^+^ cells within the total spleen. (C) Scatter plot depicting the percentage of PD1^+^CXCR5^+^ T_FH_ cells within the total CD4^+^ cell population. (D) Scatter plot depicting the percentage of CD95^+^GL7^+^ germinal center cells within the total B cell population. Each dot represents data from an individual mouse. All animals are naive unimmunized.
**Figure S4.** Freshly isolated Helios^+^CD8 Tregs do not produce cytokine. Splenocytes were harvested from young (2–3 months), middle‐aged (10–12 months) and old‐aged mice (20–24 months) for (A) or from middle‐aged mice for (B). (A) Cells were then stimulated for 4 h with or without PMA/ionomycin in the presence of brefeldin A. Representative FACS showing the production of TNF‐α and IFN‐γ in Ly49^−^ (gray), Ly49^+^Hellios^−^ (black), and Ly49^+^Helios^+^ cells (ruby). (B) Freshly isolated splenocytes were stimulated for 4 h with αCD3/CD28 in the presence of brefeldin A. Left, TNF‐α production in total Ly49^−^CD8^+^ non‐Tregs; Right, IL‐10 production in CD8^+^ Tregs. Each pair of symbols represents the results from an individual mouse. The *p*‐values were calculated by paired Student's *t*‐test.
**Figure S5.** Aged CD8^+^ Tregs exhibit comparable suppressive functions in vivo. (A) Experimental setup for (B) to (F). Two‐month‐old DKO mice received either young WT CD8^+^ Tregs or middle‐aged CD8^+^ Tregs. Host mice and donor T cells were examined 3 months later. (B) Representative FACS profiles of pregated splenic B cells to show the spontaneous GC responses. (C) The percentage of GC B cells is shown. (D) The percentage of donor CD8^+^ Tregs in the total CD8^+^ Treg gate (Ly49^+^CD122^+^) is shown. (E) Representative FACS profiles of pregated Ly49^+^CD122^+^ CD8^+^ T cells to show Helios expression. (F) The percentage of Helios^+^ cells in young versus middle‐aged donors is shown. (G) Experimental setup for (H). (H) Day 14 post‐LCMV infection, the ratio of CD4 T_FH_ versus Helios^+^CD8^+^ Tregs is shown. Each symbol represents the results of an individual mouse. The *p*‐values were calculated by one‐way ANOVA or Student's *t*‐test.
**Figure S6.** Enrichment of NK signatures in CD8^+^ Tregs isolated from middle‐aged donors. GSEA analysis was performed on the bulk RNA‐seq results presented in Figure 7. Left, the enrichment of NK_UP signature; Right, the enrichment of NK_Down signature. Red, young CD8^+^ Treg; Purple, middle‐aged CD8^+^ Treg.
**Figure S7.** NKG2D is not apparently involved in CD8^+^ Tregs in young animals and in vitro. The splenocytes isolated from 6‐ to 8‐week‐old mice were examined by FACS. (A) Representative FACS profiles of pregated CD8^+^ Tregs are shown. (B) MFI of NKG2D on pregated Helios^+^CD8^+^ Tregs. (C) The frequency of Helios^+^CD8^+^ Tregs in total CD8^+^ T cells is shown. (D) The frequency of Helios^+^CD8^+^ Tregs in total spleen is shown. (E) MFI of Helios in pregated Helios^+^CD8^+^ Tregs. (F) Experimental setup for (G). (G) Post 7 days of culture, left, the percentage of Helios^+^ cells in live CD8^+^; middle, MFI of Helios in pregated Helios^+^CD8^+^ Tregs; and right, MFI of granzyme B in pregated Helios^+^CD8^+^ Tregs. Each symbol in B to E and each pair of symbols in G represent the results from an individual mouse. The *p*‐values were calculated by Student's *t*‐test (N.S., not significant, ****p < 0.0001).

## Data Availability

Bulk RNA‐seq data are openly available in a public repository and are accessible at GEO: GSE252718. Other data that support the findings of this study are available from the corresponding author upon reasonable request.
