# Supplementary material for: Age‐Dependent Bi‐Phasic Dynamics of Ly49+ CD8 + Regulatory T Cell Population
Source: Aging Cell. 2024 Dec 18;24(4):e14461. doi: 10.1111/acel.14461 (PMC11984669; doi:10.1111/acel.14461)
Supplement: Supplementary file 1 — Figure S1. TGF‐β induces Helios, but not Ly49 expression during in vitro CD8+ T cell activation. Purified naive OT‐1 TCR transgenic CD8+ T cells were activated by anti‐CD3/CD28 in the presence of 10μg/ml TGF‐β‐neutralizing antibody (clone#1D11.16.8, BioXcell) or 2.5ng/ml hTGF‐β1 (BioLegend). Two days later, live CD8+ T cells were FACS sorted and analyzed by bulk RNA‐seq. The mRNA levels for Klra6 (A), Klra7 (B), and Ikzf2 (C) are shown. Each symbol represents the results from an independent culture sample. The p‐values were calculated by Student's t‐test. Figure S2. Age distribution of CD44hi and Ly49+ TVM cells. (A) Number of CD8+ Tregs in the total splenocytes of young (2–3 months), middle‐aged (10–12 months), and old‐aged mice (20–24 months). The statistical significance of the data presented (means ± SEM) was determined using one‐way ANOVA (*p < 0.05, **p < 0.01). (B) Scatter plot of CD44highCD8+ (top) and Ly49−CD122hiCD49dlow TVM cells (bottom) as a percentage of total CD8+ cells across a 2‐ to 28‐month age range, with individual data points for male (blue) and female (red). Each dot represents an individual mouse. Figure S3. Age‐related kinetics of CD4+ Treg, CD4+TFH, and germinal center B cells. (A) Scatter plot illustrating the percentage of Foxp3+ cells within the total CD4+ cell population across a 0‐ to 27‐month age range. (B) Scatter plot showing the percentage of CD4+Foxp3+ cells within the total spleen. (C) Scatter plot depicting the percentage of PD1+CXCR5+ TFH cells within the total CD4+ cell population. (D) Scatter plot depicting the percentage of CD95+GL7+ germinal center cells within the total B cell population. Each dot represents data from an individual mouse. All animals are naive unimmunized. Figure S4. Freshly isolated Helios+CD8 Tregs do not produce cytokine. Splenocytes were harvested from young (2–3 months), middle‐aged (10–12 months) and old‐aged mice (20–24 months) for (A) or from middle‐aged mice for (B). (A) Cells were then stimulated [file ACEL-24-e14461-s001.docx]

**Supplemental Figures**

**Title:** Age-dependent bi-phasic dynamics of Ly49^+^CD8^+^ regulatory T cell population

**Authors:** Saranya Srinivasan^1^, Shruti Mishra^1,2^, Kenneth Ka-Ho Fan^1^, Liwen Wang^1,3^, John Im^1^, Courtney Segura^1^, Neelam Mukherjee^4^, Gang Huang^5^, Manjeet Rao^5^, Chaoyu Ma^1,*^, and Nu Zhang^1,6, *^


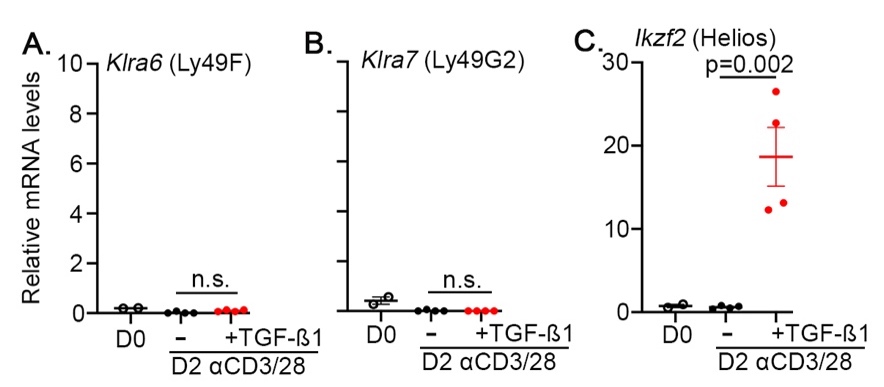


**Figure S1. TGF-β induces Helios, but not Ly49 expression during in vitro CD8^+^ T cell activation**.

Purified naïve OT-1 TCR transgenic CD8^+^ T cells were activated by anti-CD3/CD28 in the presence of 10μg/ml TGF-β neutralizing antibody (clone#1D11.16.8, BioXcell) or 2.5ng/ml hTGF-β1 (BioLegend). Two days later, live CD8^+^ T cells were FACS sorted and analyzed by bulk RNA-seq. The mRNA levels for *Klra6* (**A**), *Klra7* (**B**) and *Ikzf2* (**C**) are shown. Each symbol represents the results from an independent culture sample. The p-values were calculated by Student *t*-test.


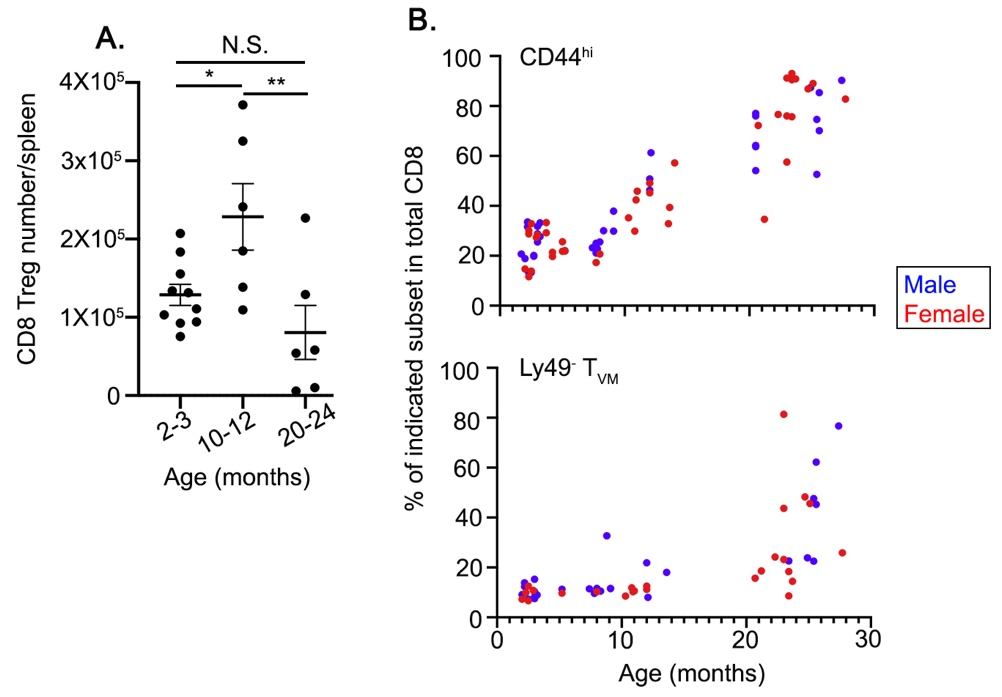


**Figure S2. Age distribution of CD44^hi^ and Ly49^+^ T_VM_ cells.**

**(A)** Number of CD8^+^ Tregs in the total splenocytes of young (2-3 months), middle-aged (10-12 months), and old-aged mice (20-24 months). The statistical significance of the data presented (means ± SEM) was determined using one-way ANOVA (*p < 0.05, **p < 0.01). **(B)** Scatter plot of CD44^high^CD8^+^ (top) and Ly49^-^CD122^hi^CD49d^low^ T_VM_ cells (bottom) as a percentage of total CD8^+^ cells across a 2–28-month age range, with individual data points for male (blue) and female (red). Each dot represents an individual mouse.

**
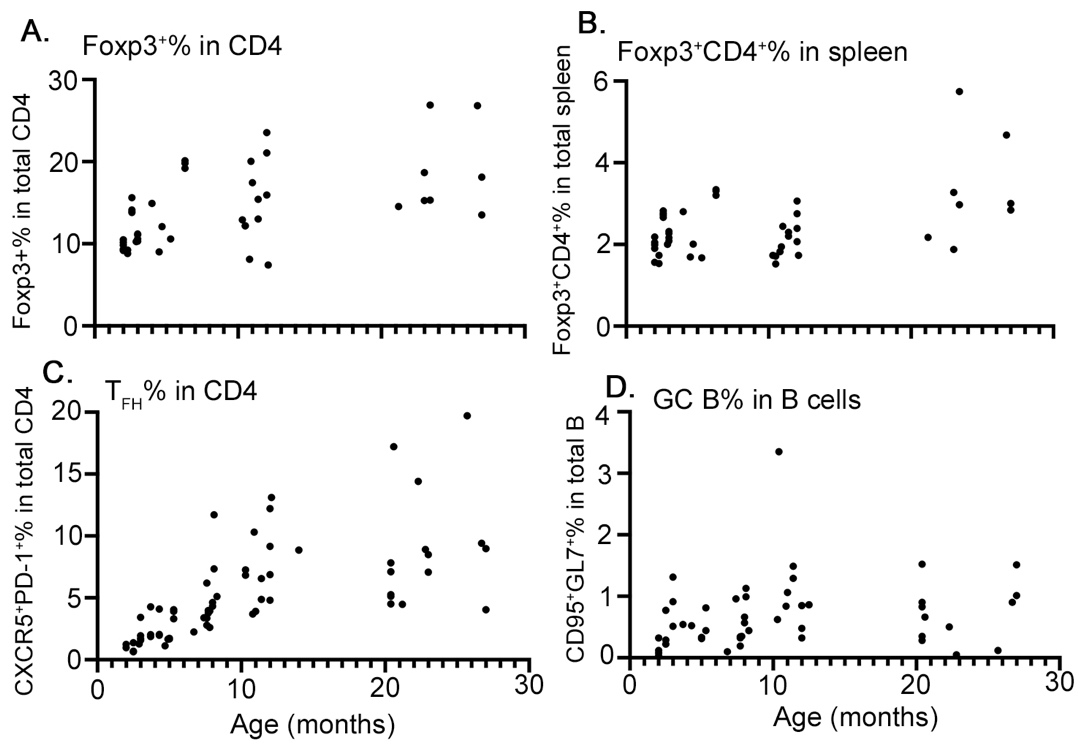
**

**Figure S3. Age-related kinetics of CD4^+^ Treg, CD4^+^T_FH_ and germinal center B cells.** **(A)** Scatter plot illustrating the percentage of Foxp3^+^ cells within the total CD4^+^ cell population across a 0–27-month age range. **(B)** Scatter plot showing the percentage of CD4^+^Foxp3^+^ cells within the total spleen. **(C)** Scatter plot depicting the percentage of PD1^+^CXCR5^+^ T_FH_ cells within the total CD4^+^ cell population. **(D)** Scatter plot depicting the percentage of CD95^+^GL7^+^ germinal center cells within the total B cell population. Each dot represents data from an individual mouse. All animals are naïve unimmunized.


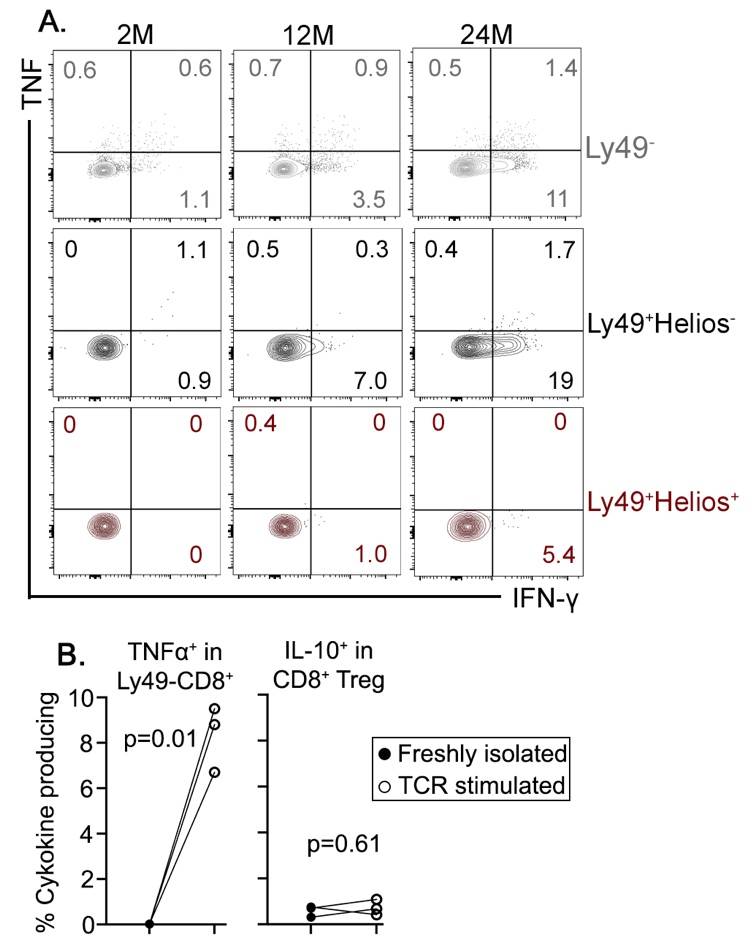


**Figure S4. Freshly isolated Helios^+^CD8 Tregs do not produce cytokine.**

Splenocytes were harvested from young (2-3 months), middle-aged (10-12 months) and old-aged mice (20-24 months) for (A) or from middle-aged mice for (B). (**A**) Cells were then stimulated for 4 hrs with or without PMA/ionomycin in the presence of brefeldin A. Representative FACS showing the production of TNF-α and IFN-γ in Ly49^‑^ (grey), Ly49^+^Hellios^-^ (black) and Ly49^+^Helios^+^ cells (ruby). (**B**) Freshly isolated splenocytes were stimulated for 4 hrs with αCD3/CD28 in the presence of brefeldin A. Left, TNF-α production in total Ly49^-^CD8^+^ non-Tregs; Right, IL-10 production in CD8^+^ Tregs. Each pair of symbols represents the results from an individual mouse. The *p*-values were calculated by paired Student *t*-test.


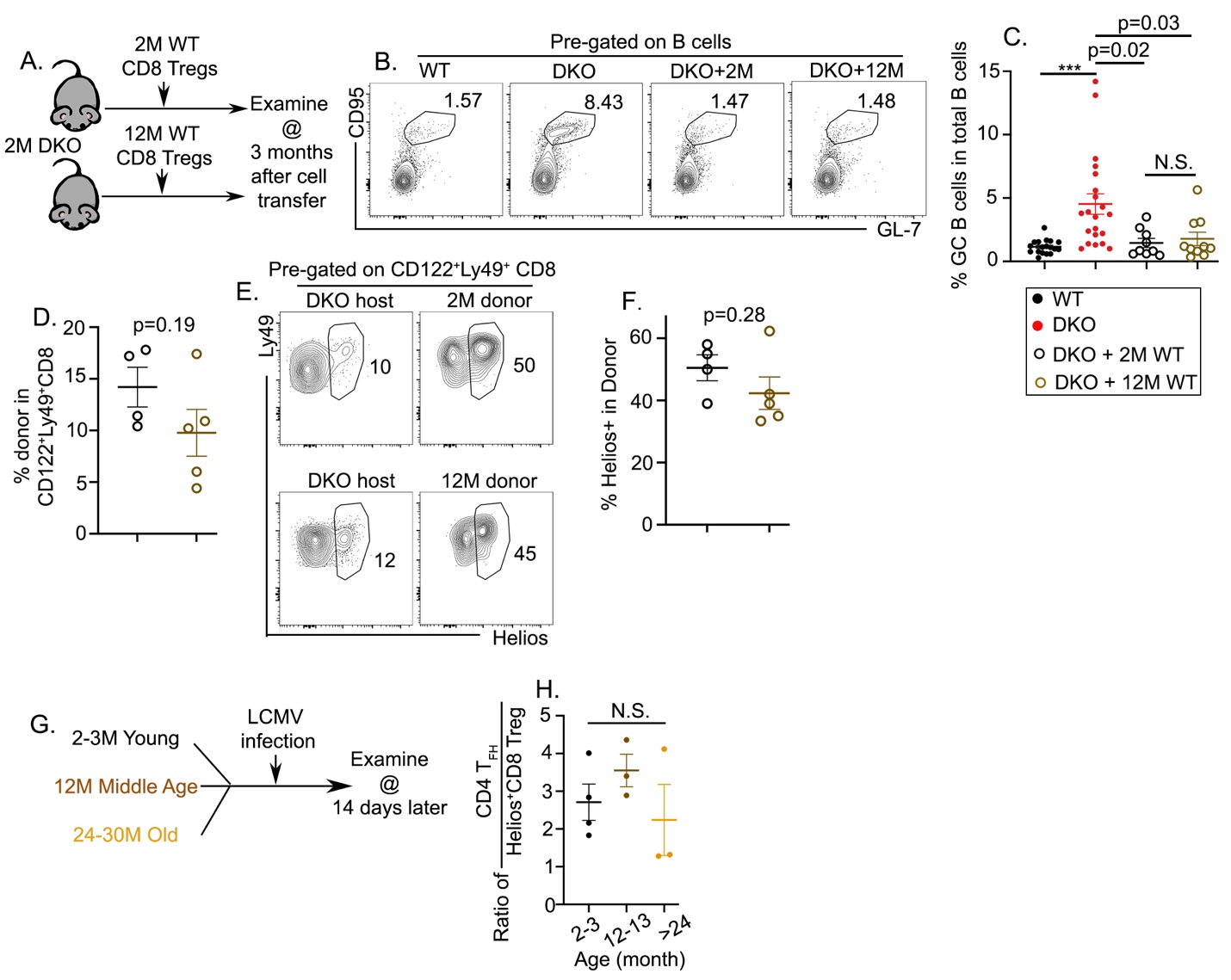


**Figure S5. Aged CD8^+^ Tregs exhibit comparable suppressive functions in vivo.**

(**A**) Experimental setup for (B) to (F). 2-month-old DKO mice received either young WT CD8^+^ Tregs or middle-aged CD8^+^ Tregs. Host mice and donor T cells were examined three months later. (**B**) Representative FACS profiles of pre-gated splenic B cells to show the spontaneous GC responses. (**C**) The percentage of GC B cells is shown. (**D**) The percentage of donor CD8^+^ Tregs in the total CD8^+^ Treg gate (Ly49^+^CD122^+^) is shown. (**E**) Representative FACS profiles of pre-gated Ly49^+^CD122^+^ CD8^+^ T cells to show Helios expression. (**F**) The percentage of Helios^+^ cells in young vs middle-aged donors is shown. (**G**) Experimental setup for (H). (**H**) Day 14 post-LCMV infection, the ratio of CD4 T_FH_ vs Helios^+^CD8^+^ Tregs is shown. Each symbol represents the results of an individual mouse. The p-values were calculated by one-way ANOVA or Student *t*-test.


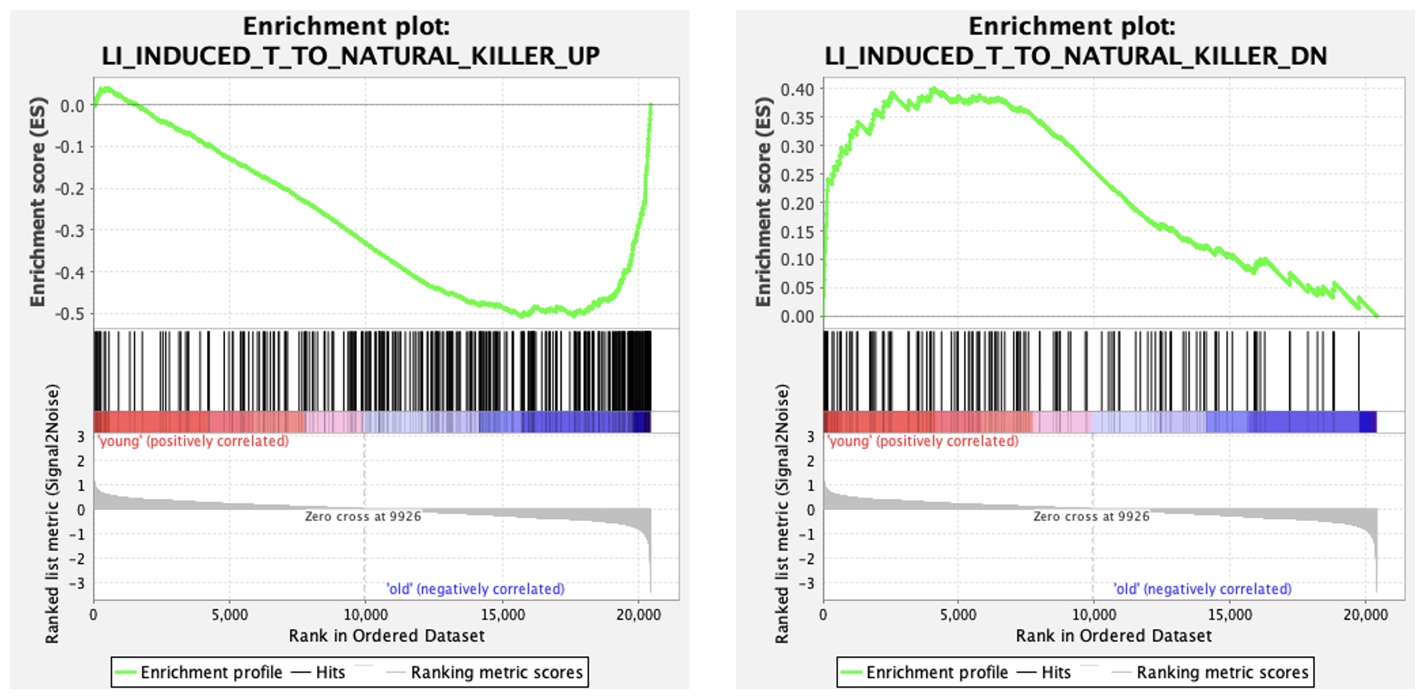


**Figure S6. Enrichment of NK signatures in CD8^+^ Tregs isolated from middle-aged donors.**

GSEA analysis was performed on the bulk RNA-seq results presented in Figure 7. Left, the enrichment of NK_UP signature; Right, the enrichment of NK_Down signature. Red, young CD8^+^ Treg and Purple, middle-aged CD8^+^ Treg.


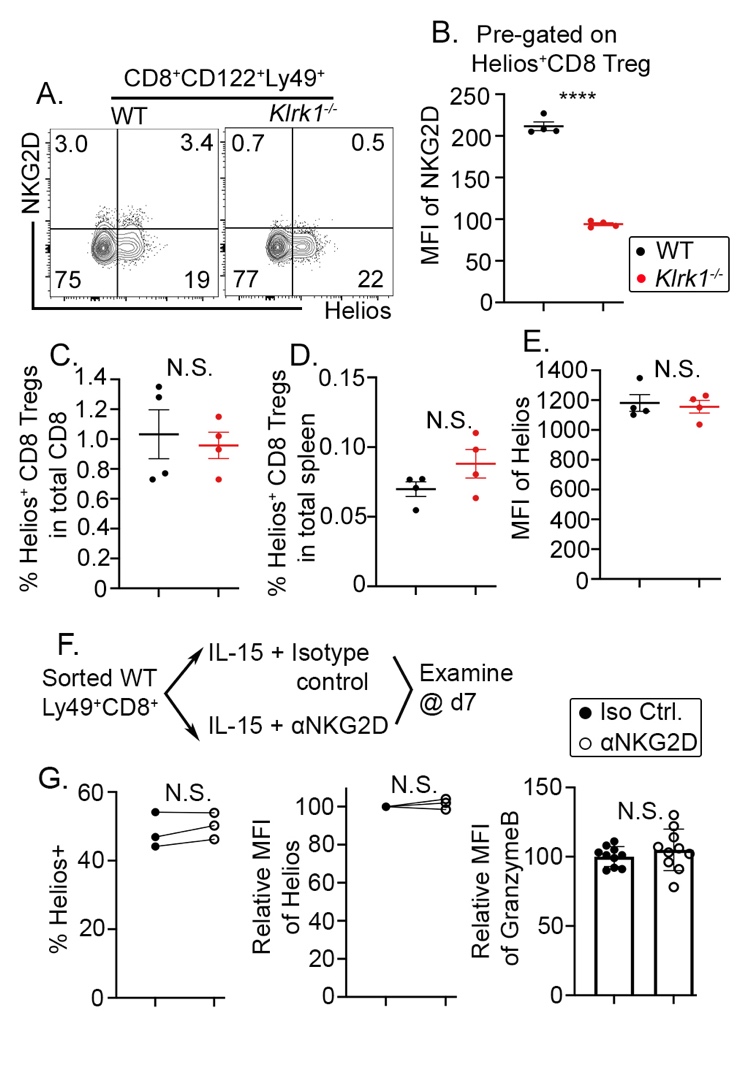


**Figure S7. NKG2D is not apparently involved in CD8^+^ Tregs in young animals and in vitro.**

The splenocytes isolated from 6-8 weeks-old mice were examined by FACS. (**A**) Representative FACS profiles of pre-gated CD8^+^ Tregs are shown. (**B**) MFI of NKG2D on pre-gated Helios^+^CD8^+^ Tregs. (**C**) The frequency of Helios^+^CD8^+^ Tregs in total CD8^+^ T cells is shown. (**D**) The frequency of Helios^+^CD8^+^ Tregs in total spleen is shown. (**E**) MFI of Helios in pre-gated Helios^+^CD8^+^ Tregs. (**F**) Experimental setup for (G). (**G**) Post seven days of culture, left, the percentage of Helios^+^ cells in live CD8^+^; middle, MFI of Helios in pre-gated Helios^+^CD8^+^ Tregs; right, MFI of granzyme B in pre-gated Helios^+^CD8^+^ Tregs. Each symbol in B to E and each pair of symbols in G represent the results from an individual mouse. The p-values were calculated by Student *t*-test (N.S., not significant, ****, p<0.0001).
